# Doses for X‐ray and electron diffraction: New features in RADDOSE‐3D including intensity decay models

**DOI:** 10.1002/pro.5005

**Published:** 2024-06-25

**Authors:** Joshua L. Dickerson, Patrick T. N. McCubbin, Jonathan C. Brooks‐Bartlett, Elspeth F. Garman

**Affiliations:** ^1^ Department of Biochemistry, Dorothy Crowfoot Hodgkin Building University of Oxford Oxford UK; ^2^ MRC Laboratory of Molecular Biology Cambridge Biomedical Campus Cambridge UK; ^3^ Division of Structural Biology, Nuffield Department of Medicine University of Oxford Oxford UK

**Keywords:** diffraction‐decay weighted dose, dose, graphical user interface, intensity decay model, MicroED, RADDOSE‐3D, radiation damage

## Abstract

New features in the dose estimation program RADDOSE‐3D are summarised. They include the facility to enter a diffraction intensity decay model which modifies the “Diffraction Weighted Dose” output from a “Fluence Weighted Dose” to a “Diffraction‐Decay Weighted Dose”, a description of RADDOSE‐ED for use in electron diffraction experiments, where dose is historically quoted in electrons/Å^2^ rather than in gray (Gy), and finally the development of a RADDOSE‐3D GUI, enabling easy access to all the options available in the program.

## 1 INTRODUCTION

Structural biology has until recently relied on X‐ray crystallography to provide much of the three‐dimensional information on proteins and other macromolecules that inform biological function. Recently, single‐particle cryogenic electron microscopy and electron diffraction techniques have advanced to the point where results from them are also giving new and exciting contributions to our knowledge. However, in all of these experimental methods, the samples suffer from radiation damage (RD) inflicted by the incident X‐rays/electrons, and this RD remains one of the major bottlenecks to accurate structure determination. RD in macromolecular crystallography (MX) has been characterised over the last 60 years (for a recent review see Garman & Weik, 2023) and manifests in both reciprocal space and in real space. In reciprocal space, there is fading of the diffracted signal, starting with the highest resolution reflections and gradually extending inwards to lower resolution as irradiation continues. Finding an appropriate model for this intensity decay has proved challenging and this issue is addressed in more detail below. Diffraction fading ultimately affects the biological detail that can be gleaned from the structure, so it has become a mainstream concern for MX. Concomitant with the reflection intensity decrease, for cryo‐cooled crystals at a synchrotron, the unit cell volume is seen to expand, the scaling *B*‐factors increase linearly with exposure, the internal agreement quality indicators for the dataset become worse (e.g., higher *R* 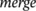 values), and the mosaicity often increases. In real space, atomic *B*‐factors become larger, and specific structural damage to particular moieties is observed in a reproducible order; for example, reduction of metal ions and disulphide bond scission (also observed at room temperature) occur before decarboxylation of aspartate and glutamate residues.

**FIGURE 1:**
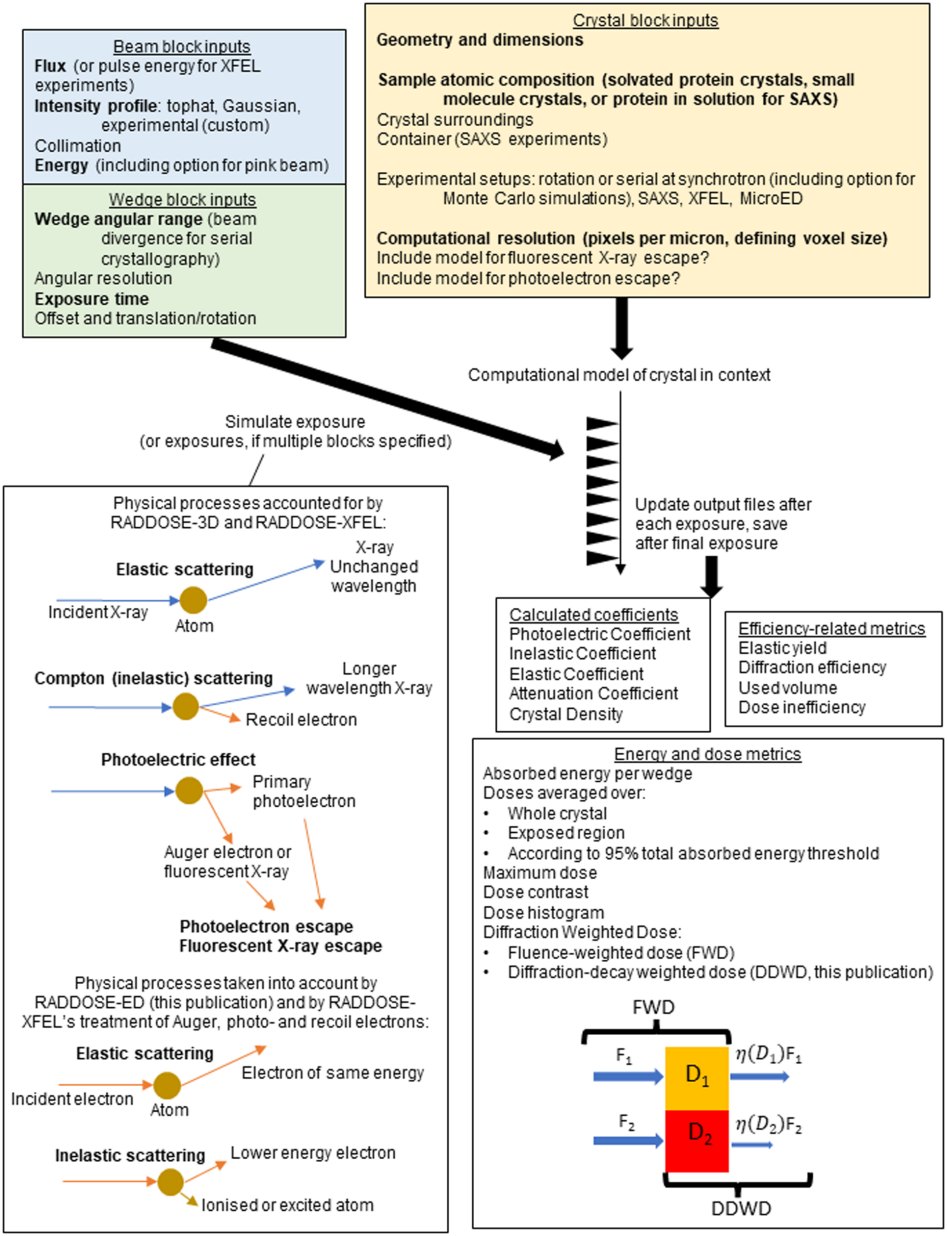
Overview of how RADDOSE‐3D is structured to take inputs describing the crystal (or solution sample for SAXS), beam, and exposure, and then to output a range of metrics relating to the diffraction efficiency and the dose. Required inputs are in bold, but see the RADDOSE‐3D documentation for the exact implementation and structure of the input file. A more detailed discussion concerning the interpretation of different dose metrics is included in Section 2, which describes the implementation of intensity decay models in RADDOSE‐3D. In the figure, $D_{1}$ and $D_{2}$ refer to the absorbed dose at two different positions in a crystal, see Section 2 for more details. Section 3 describes RADDOSE‐ED, which together with Dickerson et al. (2020), implements processes involving the interaction of electrons with atoms.

In MX, the primary metric against which the rates of damage have been monitored is the absorbed dose, defined as the absorbed energy (J) per mass (kg) in units of gray (Gy = J/kg). The dose in an experiment cannot be measured, it can only be estimated from the properties of the beam (incident beam flux density, beam profile, and energy) and the sample (atomic composition, dimensions of the crystal) so that the absorption coefficients can be calculated. To enable experimenters to more easily estimate dose, we have written and freely distributed an open‐source software program called RADDOSE‐3D (Bury et al., 2018; Zeldin, Gerstel, & Garman, 2013) which allows time‐ and space‐resolved modelling of dose. Due largely to the initial object‐orientated modular architecture of the code, we have been able to continually develop and improve it for the last 11 years. In RADDOSE‐3D, an experiment is represented by three objects, the “Crystal”, “Beam”, and “Wedge” blocks. By defining these objects in the program input, RADDOSE‐3D can “simulate” the experiment and estimate the absorbed dose within the sample (Figure 1). A full description of progress from the first release in 2013 until 2018 was given by Bury et al. (2018). Other papers since then have detailed various extensions to the code and RADDOSE‐3D can now be used to estimate the absorbed dose for a wide range of structural biology modalities. Specifically, modifications to the code have been implemented which allow dose estimations for small angle X‐ray scattering (SAXS) investigations (Brooks‐Bartlett, 2016) and for small molecule crystallography experiments (Christensen et al., 2019), with a subsequent improvement to include the energy carried away from the sample by fluorescent photons (Fernando et al., 2021). A more sophisticated treatment of photoelectron escape (which reduces the absorbed energy and thus lowers the dose) has been implemented to cater for the increased use of microbeams and microcrystals, and now includes an option for Monte Carlo simulations to provide more accurate calculations (Dickerson & Garman, 2021). We have also released RADDOSE‐XFEL (Dickerson et al., 2020) which can provide estimates of the dose absorbed during very short X‐ray pulse (fs) experiments at X‐ray free electron lasers (XFELs) by tracking the time taken for the various energy loss processes.

In this article, we will summarise the unpublished capabilities recently implemented in RADDOSE‐3D. We divide the descriptions of new work into three main sections below: (1) the option to specify an intensity decay model, (2) a description of RADDOSE‐ED for use in microcrystal electron diffraction (MicroED) studies where traditionally the effects of RD have been monitored against fluence (electrons/area, e^−^/Å^2^) rather than against dose in gray, and (3) an introduction to a new graphical user interface (GUI) that gives researchers all the current capabilities in a user‐friendly form.

However, to ensure clarity between the different fields involved in the descriptions below, here we provide unambiguous definitions of certain key terms. Fluence is defined as photons (or electrons) per unit area (ph/mm^2^ or e^−^/Å^2^), respectively, flux is photons (or electrons) per second, and flux density is photons (or electrons) per unit area per second. The information coefficient (or “diffraction efficiency” for modalities involving diffraction), is defined as the signal intensity per MGy of absorbed dose. It should be noted that cryoEM papers in the literature frequently discuss quantities in terms of “per unit damage,” which often refers to global damage measured by an increase in overall *B*‐factor. In MX papers, $I/I_{0}$ (where I is the total intensity of a dataset or section of data and $I_{0}$ is the intensity of the same sweep of data extrapolated to zero dose) is used as a unit of global damage and *B*_net_ is a new unit of specific damage (Shelley & Garman, 2022).

## 2 INTENSITY DECAY MODELS IN RADDOSE‐3D

Intensity decay models (IDMs) describe the decrease in the intensity of diffracted X‐rays as the absorbed dose increases. Table 1 shows commonly used or recently proposed IDMs. The associated parameters are either purely empirical or have some physical justification (for further discussion of the general form of IDMs, see Section 2.2.1). In the first part of this section, we will demonstrate the implementation of a previously published IDM (that of Leal et al. (2013)) into RADDOSE‐3D and show how dose estimates by this implementation can explain the progression of specific and global RD in diffraction data. In the second part, we place this IDM in a broader context, show how we estimated its parameter values through fitting to diffraction data, and analyse its physical basis in order to motivate further work on how the parameter values for each crystal might be predicted from physical principles. Finally, we discuss the significance of this work for crystallographic data processing and the analysis of electron density maps.

**TABLE 1:** Intensity decay models.

Model (citation)	Form of relative intensity decay with dose	Fitted parameters	Resolution dependence of dose‐dependent decay
None	$\frac{I_{n}}{I_{0}}=1$	This is the IDM used in the original implementation of DWD as a fluence‐weighted dose (Zeldin, Brockhauser, et al., 2013).	N/A
Linear (Owen et al., 2006)	$\frac{I_{n}}{I_{0}}=1-\frac{D}{2D_{\frac{1}{2}}}$ (for $0\leq D\leq 2D_{\frac{1}{2}}$) $\frac{I_{n}}{I_{0}}=0$ (for $D>2D_{\frac{1}{2}}$)	$D_{\frac{1}{2}}$ is the half‐dose, experimentally measured at 43 MGy in Owen et al. (2006) for intensities in the resolution range 50–2.4 Å	$D_{\frac{1}{2}}$ varies with resolution shell, see discussion in Owen et al. (2006), but no explicit relationship incorporated into model.
Standard dose–response (Owen et al., 2014)	$\frac{I_{n}}{I_{0}}=\frac{I_{\infty }}{I_{0}}+\frac{1-\frac{I_{\infty }}{I_{0}}}{1+10^{\left(\log\left(x_{0}\right)-D\right)p}}$	$I_{\infty }$ is the lower asymptote (i.e., the final diffracting power). $\log\left(x_{0}\right)$ is the decay curve midpoint, p is the Hill slope.	The values of the midpoint and Hill co‐efficient may depend on the resolution.
Four‐state kinetic (Sygusch & Allaire, 1988)	$\begin{array}{l} M\left(D,h\right)=F_{\text{native}}\left(D\right)\\ +F_{\text{perturbed}}\left(D\right)+F_{\text{disordered}}\left(D,h\right) \end{array}$	$M\left(D,h\right)$ is the relative intensity for a small region of the crystal as used in Equation (3). $F_{\text{native}}$ is the contribution of the undamaged fraction of the crystal that decreases linearly with dose. $F_{\text{perturbed}}$ is the contribution of a fraction only slightly perturbed by damage (e.g., by site‐specific damage or only a few ionisation events per unit cell) such that the scattering power is still similar to the native state. $F_{\text{disordered}}$ is the contribution of a fraction of the crystal that has been significantly disordered but is still capable of contributing to diffraction. The fractions evolve according to a sequential kinetic scheme Native→Perturbed→Disordered→Amorphous with rate constants (with respect to dose) that are fitted empirically. The amorphous fraction does not contribute to diffraction and thus does not appear in the equation.	The resolution‐dependence of intensity decay is captured in the $F_{\text{disordered}}$ term.
Exponential decay (Holton, 2009; Holton & Frankel, 2010)	$\begin{array}{l} M\left(D,h\right)=\exp\left(-\frac{1}{2}B_{0}h^{2}\right)\\ \exp\left(-\ln\left(2\right)\frac{\mathrm{Dh}}{H}\right) \end{array}$	H is the Howell criterion (units MGy Å^−1^), derived from meta‐data for a range of experimental measurements, of 10 MGyÅ 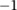 for cryo‐temperature experiments in Howells et al. (2009) based on data in resolution range 100–1 Å. $B_{0}$ is the Wilson *B*‐factor at zero dose.	$\exp\left(-\frac{1}{d}\right)$
Gaussian blurring of electron density (Atakisi et al., 2019)	$\begin{array}{l} M\left(D,h\right)=\exp\left(-\frac{1}{2}B_{0}h^{2}\right)\\ \exp\left(-\frac{{\mathrm{Dh}}^{\alpha }}{K}\right) \end{array}$	α=2 would be expected if intensity decay is due to Gaussian blurring of electron density at random locations in the crystal. However, empirical fitting suggests values of α less than 2 [18]. K is a proportionality constant. This model is equivalent to a model with linearly increasing *B*‐factor (with initial value $B_{0}$) but no dose‐dependent scale factor.	$\exp\left(-\frac{1}{d^{\alpha }}\right)$
Adapted scaling model (general form) (Beilsten‐Edmands et al., 2020; Borek et al., 2013; Evans & Murshudov, 2013; Kabsch, 2010; Leal et al., 2013)	$M\left(D,h\right)=K\exp\left(-\frac{{\mathrm{Bh}}^{2}}{2}\right)$	B is the overall *B*‐factor that increases linearly with dose. How this behaviour is implemented can vary, for example, Leal et al. (2013) suggest $B=B_{0}+\mathrm{\beta D}$ as described below, and Borek et al. (2007) suggest using a relative *B*‐factor with $B_{\mathrm{rel}}=8\pi^{2}\mathrm{cD}$ where $8\pi^{2}c$ is a constant estimated to be 1 Å^2^ MGy^−1^ (Borek et al., 2007; Kmetko et al., 2006; Krojer & von Delft, 2011) K is an empirical scale factor, sometimes denoted instead by s=1/K. Assuming no other variables are affecting the intensities (such as changes to illuminated volume), this is usually taken to be unity, as in Borek et al. (2007). This is equivalent to assuming that the effects of global damage are captured entirely by the linear *B*‐factor increase. However, Leal et al. (2013) suggest a dose‐dependent form of the scale factor $K=\exp\left(-\gamma^{2}D^{2}\right)$. This can improve the fit especially for room temperature diffraction data.	$\exp\left(-\frac{1}{d^{2}}\right)$
Adapted scaling model (as parameterized in RADDOSE‐3D, after Leal et al. (2013))	$\begin{array}{l} M\left(D,h\right)=\exp\left(-\gamma^{2}D^{2}\right)\\ \exp\left(-\frac{\left(B_{0}+\mathrm{\beta D}\right)h^{2}}{2}\right) \end{array}$	$B_{0}$ is the overall *B*‐factor at zero dose (units Å^2^), β quantifies rate of increase in overall *B*‐factor with dose (units Å^2^ MGy^−1^). γ (units MGy^−1^) determines the behaviour of the Gaussian scale factor. γ is small (≈0.04 MGy 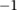 ) at cryogenic temperatures (Leal et al., 2011; Leal et al., 2013).	$\exp\left(-\frac{1}{d^{2}}\right)$

*Note*: The linear and dose–response models are given in terms of the relative intensity for the *n* ^th^ image, $\frac{I_{n}}{I_{0}}$, as a function of the cumulative dose in the *n* ^th^ image, D. For the remaining models where the resolution‐dependence of intensity decay is more precisely defined, the IDM is given as the function $M\left(D,h\right)$ in the appropriate form as to be substituted into Equation (3) below to calculate the relative diffraction efficiency for a region of a crystal. $M\left(D,h\right)$ is a function of the dose, D, and the magnitude of the scattering wavevector, $h=\frac{1}{d}$ where d is the spacing between Bragg planes. It should be noted that in practice the relative intensity is taken instead to be $\frac{I_{n}}{I_{1}}$ where $I_{1}$ is the first measured intensity at some small initial dose, so care should be taken to account for the fact that this is not the true intensity at zero dose. Similarly, care should be taken if data are normalised independently for each individual resolution bin since information on the resolution‐dependence of intensity at zero dose will be lost during this normalisation procedure.

### 2.1 Implementing an IDM in RADDOSE‐3D

In this part, we first describe how, through the incorporation of the IDM proposed by Leal et al. (2013), the diffraction‐weighted dose (DWD) metric of RADDOSE‐3D has been modified from a fluence‐weighted dose (FWD) to a diffraction‐decay weighted dose (DDWD). We then show how the DDWD estimated by RADDOSE‐3D can explain the extent of RD in electron density maps, using the dataset collected by de la Mora et al. (2020) as an example. Finally, we discuss how DDWD compares to other dose metrics that are output by RADDOSE‐3D.

#### 2.1.1 Diffraction‐weighted dose in RADDOSE‐3D

Diffraction‐weighted dose (DWD), as first implemented by Zeldin et al. (2013), weighted the cumulative dose to each part of the crystal by the incident fluence. Here we will refer to this as the fluence‐weighted dose (FWD):

(1) $$\mathrm{FWD}=\frac{\int_{t_{i-1}}^{t_{i}}\int_{\text{crystal}}D\left(\vec{x},t\right)F\left(\vec{x},t\right)d\vec{x}\mathrm{dt}}{\int_{t_{i-1}}^{t_{i}}\int_{\text{crystal}}F\left(\vec{x},t\right)d\vec{x}\mathrm{dt}}$$

where t is time, such that $t_{i-1}\to t_{i}$ describes the time of the exposure, $D\left(\vec{x},t\right)$ is the total cumulative dose (MGy) at position $\vec{x}$, and $F\left(\vec{x},t\right)$ is the flux density at position $\vec{x}$ and time t.

This is an advantageous metric compared to the total average dose across the whole crystal (weighting all voxels equally), the maximum dose (weighting the voxel with the highest dose as one, and all other voxels as zero) or the average dose in the exposed region (defining an exposed region by an incident intensity threshold and weighting all voxels in this region equally). This is because voxels that are irradiated by more intense regions of the beam contribute proportionally more to the FWD, and voxels outside the beam have negligible incident intensity and thus make negligible contribution to the FWD. However, as pointed out in the original publication (Zeldin, Brockhauser, et al., 2013) and by other studies thereafter (Brooks‐Bartlett, 2016; de la Mora et al., 2020; Warkentin et al., 2017), weighting by incident fluence alone does not account for the decay in relative intensity due to RD as the dose increases. For a true DWD, an appropriate intensity decay model (IDM) that can be applied for each volume element of the crystal must be incorporated into the definition of DWD.

#### 2.1.2 Diffraction‐decay weighted dose in RADDOSE‐3D

There is now the option to output the diffraction weighted dose result of RADDOSE‐3D as a diffraction‐decay weighted dose (Brooks‐Bartlett, 2016; de la Mora et al., 2020; Warkentin et al., 2017), DDWD, which weights the cumulative dose to each part of the crystal by the predicted fluence out of that region of the crystal. DDWD is defined as follows:

(2) $$\text{DDWD}=\frac{\int_{t_{i-1}}^{t_{i}}\int_{\text{crystal}}D\left(\vec{x},t\right)F\left(\vec{x},t\right)\eta \left(D\left(\vec{x},t\right)\right)d\vec{x}\mathrm{dt}}{\int_{t_{i-1}}^{t_{i}}\int_{\text{crystal}}F\left(\vec{x},t\right)\eta \left(D\left(\vec{x},t\right)\right)d\vec{x}\mathrm{dt}}$$

where η is the predicted relative diffraction efficiency according to the IDM. The parameters t, $F\left(\vec{x},t\right)$, and $D\left(\vec{x},t\right)$ are as defined for FWD above and η is defined according to:

(3) $$\eta =\frac{\int_{h_{\min}}^{h_{\max}}h^{2}\mathrm{IM}\left(D,h\right)\mathrm{dh}}{\int_{h_{\min}}^{h_{\max}}h^{2}\mathrm{IM}\left(0,h\right)\mathrm{dh}}$$

where $M\left(D,h\right)$ is the IDM describing the decay in relative intensity as a function of the dose D absorbed in a small volume at a certain position in the crystal, and of the magnitude of the scattering wavevector $h=\frac{1}{d}$. The integrals are evaluated using representative experimental values of $h^{2}$ and I as described in Popov and Bourenkov (2003).

The appropriate parameter values for the IDM are specified by the user in the crystal block section of the input file, as explained in the RADDOSE‐3D documentation. For the adapted scaling model (Leal et al., 2013), Equation (3) equates to:

(4) $$\eta =\exp\left(-\gamma^{2}D^{2}\right)\frac{\int_{h_{\min}}^{h_{\max}}h^{2}I\exp\left(-\frac{1}{2}\left(B_{0}+\mathrm{\beta D}\right)h^{2}\right)\mathrm{dh}}{\int_{h_{\min}}^{h_{\max}}h^{2}I\exp\left(-\frac{1}{2}B_{0}h^{2}\right)\mathrm{dh}}$$

which mirrors Equation (4) in Leal et al. (2013). See Table 1 for further explanation of $B_{0}$, β, and γ.

The representative $h^{2}$ and I values used to evaluate the integrals are those from the BEST diffraction data collected on 72 different proteins (scaled together, and with varied folds, molecular weights, space groups, and data resolutions at both cryo and room temperature) (Popov & Bourenkov, 2003). The integral is taken over all resolution shells in the BEST data (12.0–0.9 Å resolution window) and thus the DDWD output is what would be expected of a typical protein with relative intensities evaluated over this resolution window. Using representative values, rather than requiring the user to input their own [$h^{2},I\left(h\right)$] values, allows these to be coded directly into RADDOSE‐3D, thereby reducing its execution time.

The modular nature of the RADDOSE‐3D code means new models that may be proposed by the crystallography community can easily be included. If no IDM is specified, the program defaults to outputting the FWD, by setting η=1.

#### 2.1.3 Example use of RADDOSE‐3D with incorporated IDM

To validate the implementation of this DDWD in RADDOSE‐3D, we reanalysed the high dose rate, room temperature dataset from de la Mora et al. (2020). RADDOSE‐3D was first used to calculate the FWD with respect to exposure time (input parameters are shown in the middle column of Supplementary Table S2). The Python script used to generate the modelled beam profile is available on the RADDOSE‐3D GitHub repository and can straightforwardly be adapted to generate any modelled beam profile for input into RADDOSE‐3D. Appropriate parameter values for the Leal et al. (2013) IDM were estimated as described in Section 2.2.2. To give the estimated DDWD with respect to the exposure time, the implementation of the Leal et al. (2013) model in RADDOSE‐3D was then run (inputs are shown in the right column of Supplementary Table S2, which are the same inputs as for the FWD calculation, except for the specification to use the Leal et al. (2013) model with the appropriate parameter values).

**FIGURE 2:**
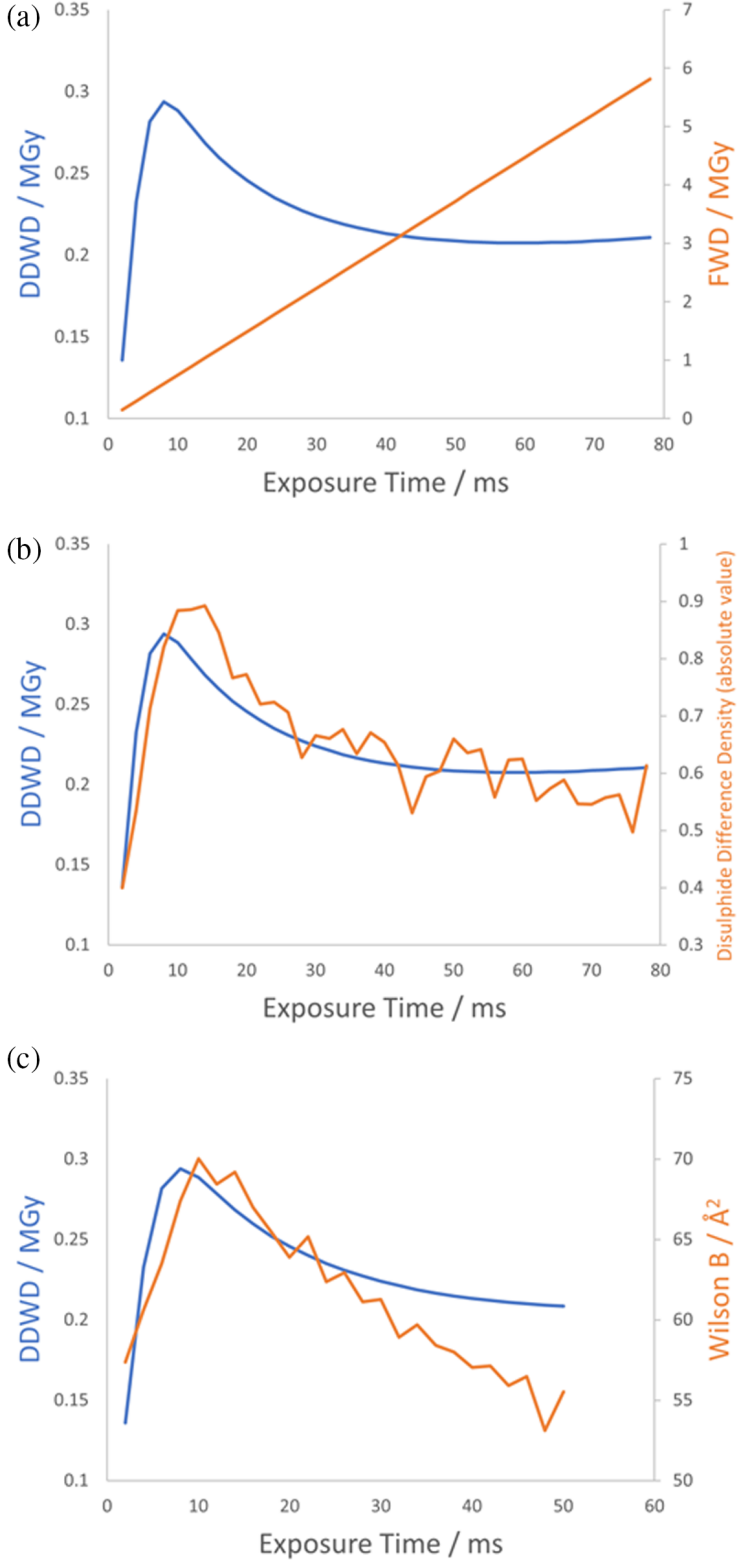
Comparison of DDWD, computed using the Leal et al. (2013) IDM implemented in RADDOSE‐3D, to the FWD computed by RADDOSE‐3D, and to measures of specific and global damage for the room temperature high dose rate diffraction data from de la Mora et al. (2020). (a) Fluence‐weighted dose (FWD, orange curve) increases linearly over the exposure time whereas diffraction‐decay weighted dose (DDWD, blue curve) goes through a maximum. (b) DDWD correlates with the observed specific damage to a disulphide bond and (c) with the Wilson *B*‐factor (see text for further details).

The results in Figure 2 show that whilst the FWD increases linearly with exposure time, DDWD increases to a maximum before gradually decreasing, because at high total doses the less damaged regions that have absorbed lower doses contribute more to the diffraction pattern. Furthermore, this behaviour correlates with the degree of damage observed in a disulphide bond: the absolute value of the integrated difference electron density for this bond is shown in Figure 2, calculated as described in de la Mora et al. (2020), where a greater value indicates a more damaged bond. DDWD gives information on the extent to which the absorbed dose manifests in the electron density map and thus correlates with the damage to this disulphide bond.

Because the integrals in Equation (3) are evaluated over the BEST data for the resolution range 12–0.9 Å, our implementation will be accurate when the resolution range of the data being analysed matches this resolution range. However, Figure 2 shows that even if the resolution ranges do not match exactly, the resultant error is systematic such that the calculated DDWD is still useful semi‐quantitatively for understanding the dose effects that manifest in the electron density map. What is critical for this analysis is that the IDM fits the relative intensity decay curve well across the full range of doses analysed in the experiment. In the original analysis in the supplementary material of de la Mora et al. (2020), an exponential decay IDM is used and the calculated DDWD significantly increases again at the highest doses, implying that the disulphide bond damage should increase again, but this behaviour is not observed in the electron density.

#### 2.1.4 Interpreting dose metrics output by RADDOSE‐3D

Accurate dose estimation is important for designing a data collection strategy. For rotation crystallography, it is necessary to ensure a full crystal rotation of data are collected before RD has significantly affected the signal. Since IDMs are all smoothly decreasing functions, often well‐approximated by the simple linear model at low/medium doses for cryo‐temperature experiments, the original implementation of DWD as the FWD (Zeldin, Brockhauser, et al., 2013) output by RADDOSE‐3D remains a useful dose metric for designing a data collection strategy; RADDOSE‐3D is implemented at multiple beamlines (such as I04 at DLS, BL12‐1 and BL12‐2 at SSRL (Garman & Weik, 2023)). Furthermore, dose estimates are essential inputs for dedicated programs that optimise data collection strategy. For example, the program BEST (Bourenkov & Popov, 2010) (within the EDNA framework (Incardona et al., 2009)) implements the Leal et al. (2013) IDM alongside a model for radiation‐induced non‐isomorphism, taking dose rate estimates from RADDOSE version 1 (Murray et al., 2004), to design an optimal data collection strategy based on a few initial diffraction images. Similarly, the program KUMA within the ZOO framework (Hirata et al., 2019) implements RADDOSE version 2 (Paithankar & Garman, 2010) to suggest exposure conditions with an absorbed dose of 10MGy (Hirata et al., 2019). For optimisation programs that implement an IDM internally, such as BEST, if it is necessary to input a single dose metric then the FWD (Zeldin, Brockhauser, et al., 2013) is the output from RADDOSE‐3D that is generally applicable for more complex exposure schemes (e.g., helical) as discussed in Bury et al. (2018). However, the behaviour of DDWD shows the advantage, particularly for room temperature data collection, of explicitly accounting for the spatial distribution of dose within the crystal and applying the model of RD to each small region of the crystal (see Section 2.2.2 for a related discussion on using the FWD to fit IDMs). Among the outputs of RADDOSE‐3D is a file containing the dose for each voxel of the crystal (Bury et al., 2018).

A second important application for dose estimation is to understand the extent of RD in electron density maps. It is essential to avoid the misinterpretation of radiation‐damage‐induced changes as biologically significant. DDWD is more informative than FWD about the extent of RD in the final electron density map, as illustrated by the analysis in Section 2.1.3.

When evaluating different dose metrics, it is important to account for how robust the dose metric is to inaccuracies in the model for the beam intensity profile, particularly for the low‐intensity edges of the beam. For example, the average dose in the exposed region is sensitive to such inaccuracies, whereas FWD and DDWD are relatively insensitive to them. Another strategy is to make a conservative approximation of the beam as purely Gaussian (i.e., neglecting any low‐intensity tails), as discussed in de la Mora et al. (2020).

### 2.2 Understanding the IDM implemented in RADDOSE‐3D

In this part we first place the IDM proposed by Leal et al. (2013) and implemented in RADDOSE‐3D in the context of some general properties of all IDMs (Section 2.2.1), then show how this IDM was fitted to diffraction data to obtain parameter values for the DDWD calculation in Section 2.1.3 (Section 2.2.2), and finally explore the physical basis for the terms of this IDM (Sections 2.2.3 and 2.2.4) to motivate further work towards an IDM with predictable parameter values.

#### 2.2.1 Introduction to the general form of IDMs

IDMs all contain an implicit assumption of uniform irradiation of the crystal or region of the crystal to which the IDM applies. To achieve uniform irradiation in practice in experimental studies, it is possible to use a beam with an exceptionally flat “top hat” intensity profile, such as is implemented at the EMBL beamline, P14, at PETRA III, Hamburg (Garman & Weik, 2017). Alternatively, a more common approach is to apply an appropriate correction for non‐uniform illumination during analysis, for example, the three‐beam model in the supplementary material of de la Mora et al. (2020) which combines a model for the beam and the exponential “H‐model” IDM (see next paragraph) (Holton & Frankel, 2010) into a single model. In the definition of DDWD, the IDM is applied to many small regions of the crystal, each of which is small enough to be treated as uniformly irradiated. The comparative advantage of using RADDOSE‐3D for the analysis of IDMs is that it directly calculates the impact of non‐uniform illumination on the distribution of dose in the crystal (Bury et al., 2018).

**FIGURE 3:**
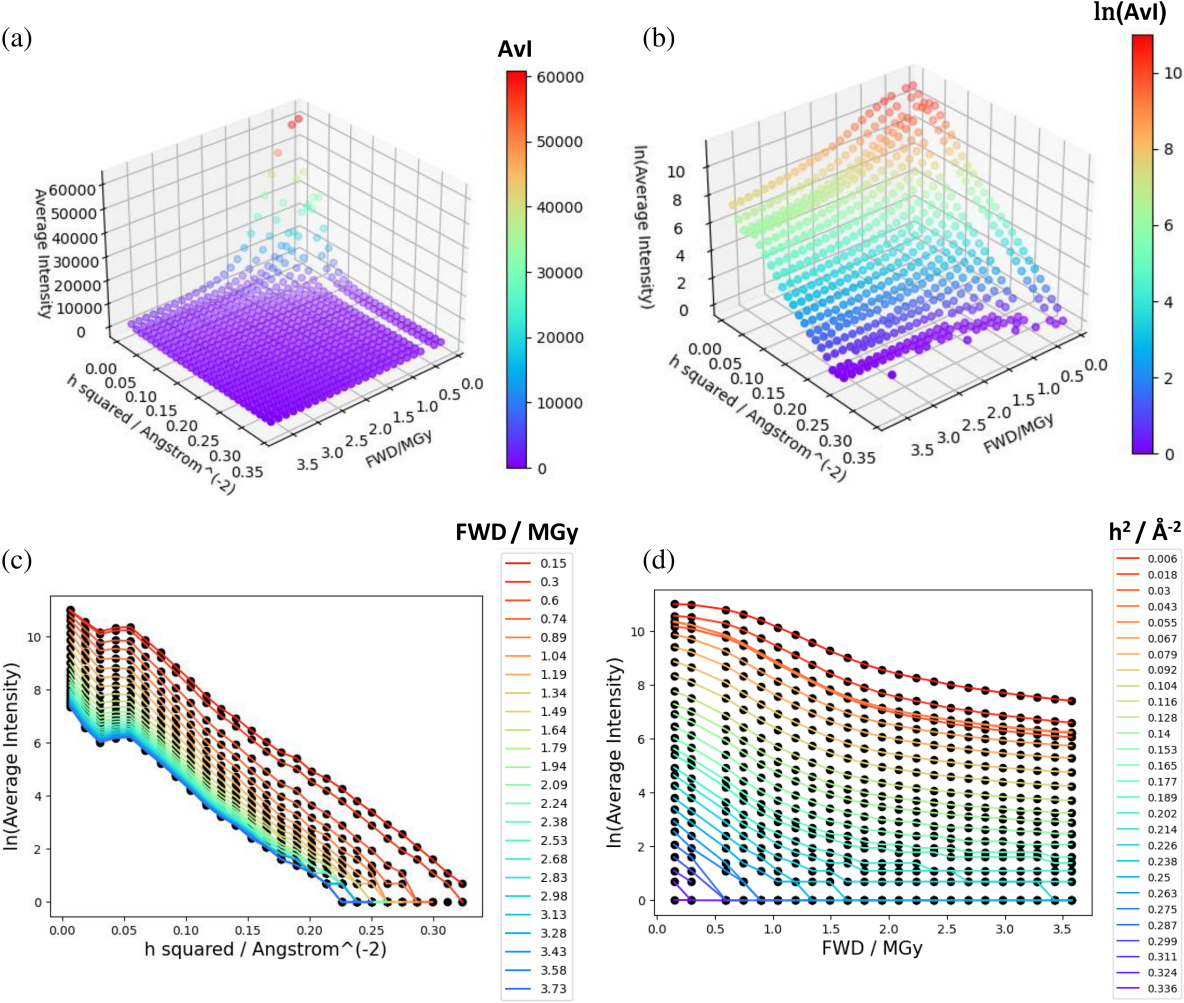
Average intensity as a function of dose and resolution, illustrated for merged but not scaled reflection room temperature high‐dose rate data from de la Mora et al. (2020). FWD was calculated by RADDOSE‐3D as described in Supplementary Table S2 and averaging by thin resolution shells (evenly spaced $h^{2}$) was performed by AIMLESS. The FWD is for the cumulative exposure time (e.g., 30 ms) whereas the average intensities are for an individual exposure (always 2 ms). An anomalously weak exposure at 0.45 MGy was removed from the data before plotting. (a) is a plot of the average intensity (AvI) against FWD and $h^{2}$ coloured by intensity value, (b) is a plot of the natural logarithm of the average intensity (ln(AvI)) against FWD and $h^{2}$ coloured by ln(AvI), (c) is a plot of ln(AvI) against $h^{2}$ coloured by FWD value and (d) is a plot of ln(AvI) against FWD coloured by $h^{2}$ value.

Figure 3 shows typical diffraction intensity data from the room‐temperature high dose rate dataset described in de la Mora et al. (2020). The data are plotted as the average intensity for a series of small resolution shells, for each of a series of sequential small exposures (for this dataset, an exposure time of 2 ms per exposure). The figure demonstrates that intensity decay depends on both dose and resolution. The definition of the relative diffraction efficiency η, Equation (3), encodes this dependence on dose and resolution. To account for the fact that spherically averaged squared structure‐factor magnitudes are a complicated function of resolution, Equation (3) uses the empirical approximation given by the BEST data (Popov & Bourenkov, 2003). However, it is important to stress that these data do not include the effect on intensity of the atomic *B*‐factors at zero dose, so this effect must be encoded into $M\left(D,h\right)$. The simplest way to do this is through a term $\exp\left(-\frac{1}{2}B_{0}h^{2}\right)$ where $B_{0}$ is an average isotropic *B*‐factor at zero dose (using an average *B*‐factor assumes the distribution of atomic *B*‐factors is not too broad or skewed). $M\left(D,h\right)$ includes further terms that describe the dose‐dependence of intensity decay. For example, a “scale” term describes any resolution‐independent contribution to intensity decay (such as $K=\exp\left(-\gamma^{2}D^{2}\right)$ within the model of Leal et al. (2013). A final term describes dose‐dependent intensity decay where the decay varies depending on the resolution. The two main hypotheses for this term are the “B‐model” $\exp\left(-\frac{1}{2}\beta {\mathrm{Dh}}^{2}\right)$, as suggested in the Leal et al. model (Leal et al., 2013) and other scaling models, and the “H‐model” $\exp\left(-\ln\left(2\right)\frac{1}{H}\mathrm{Dh}\right)$ (Holton & Frankel, 2010) (see Table 1 for further citations, parameter definitions, and units).

The evaluation of IDMs requires meta‐analyses of many datasets to increase the statistical power of the hypothesis testing. This approach has been implemented multiple times (Atakisi et al., 2019; Holton & Frankel, 2010; Howells et al., 2009; Leal et al., 2013). The resolution‐dependence of IDMs is especially significant because the loss of diffraction efficiency in the higher resolution shells has implications for the ability of the structure to inform biological hypotheses (Owen et al., 2006). An advantage of the B‐model is that it directly encodes the robust linear relationship observed between scaling *B*‐factor and dose (Borek et al., 2010; Bourenkov & Popov, 2010; Kmetko et al., 2006; Leal et al., 2013). A linear increase of *B*‐factor as dose increases is expected under a central limit theorem if radiation‐induced atomic displacements are randomly distributed, small but numerous, and accumulate in proportion to the dose (Borek et al., 2013). It has also been suggested that a different resolution‐dependence, and thus form of IDM, might apply at medium to high resolution (<10 Å) compared to low resolutions (>10 Å) (Atakisi et al., 2019). Central to crystallographic data analysis is the equivalence of modelling unit cell constituents as a collection of point scattering sources (i.e., atoms) versus scattering from a continuous electron density. In the context of IDMs, it has been shown that increasing the scaling *B*‐factor is an equivalent model to that of the Gaussian blurring of electron density at random locations in the unit cell (Atakisi et al., 2019).

Before we consider how the parameter values of the Leal et al. (2013) IDM were fitted for use in our DDWD calculation, it is worth emphasising that the parameter values of IDMs are temperature‐dependent. For example, the γ parameter of this IDM is approximately zero only for cryogenic datasets (Leal et al., 2013) whereas for room temperature datasets the scale factor term becomes strongly dose‐dependent. Temperature will affect not only the energy required to break bonds (and hence the rate of bond breakage per unit dose), but also the mobility of ions and radicals and their subsequent radiation chemistry, and thus the distribution of the absorbed dose both within unit cells and through the crystal (Weik & Colletier, 2010). Finally, none of the models in Table 1 explicitly account for the possibility of dose‐rate effects (see (Garman & Weik, 2023) for further discussion).

#### 2.2.2 Fitting the adapted scaling model

To estimate parameter values for use in the DDWD calculation in Section 2.1.3, the Leal et al. (2013) model was fitted to the room‐temperature, high dose rate data from de la Mora et al. (2020). Merged (but not scaled) reflection files for each sequential 2 ms exposure dataset were reanalysed: note that no *B*‐factor or scale factor correction had been applied to the intensities before this analysis. We stress that the appropriate intensities to use for fitting the model are the result of reflection integration (in this case by CrystFEL (White et al., 2012)) and thus have no contribution from background, and the intensity values tend to zero at high doses as shown in Figure 3.

**FIGURE 4:**
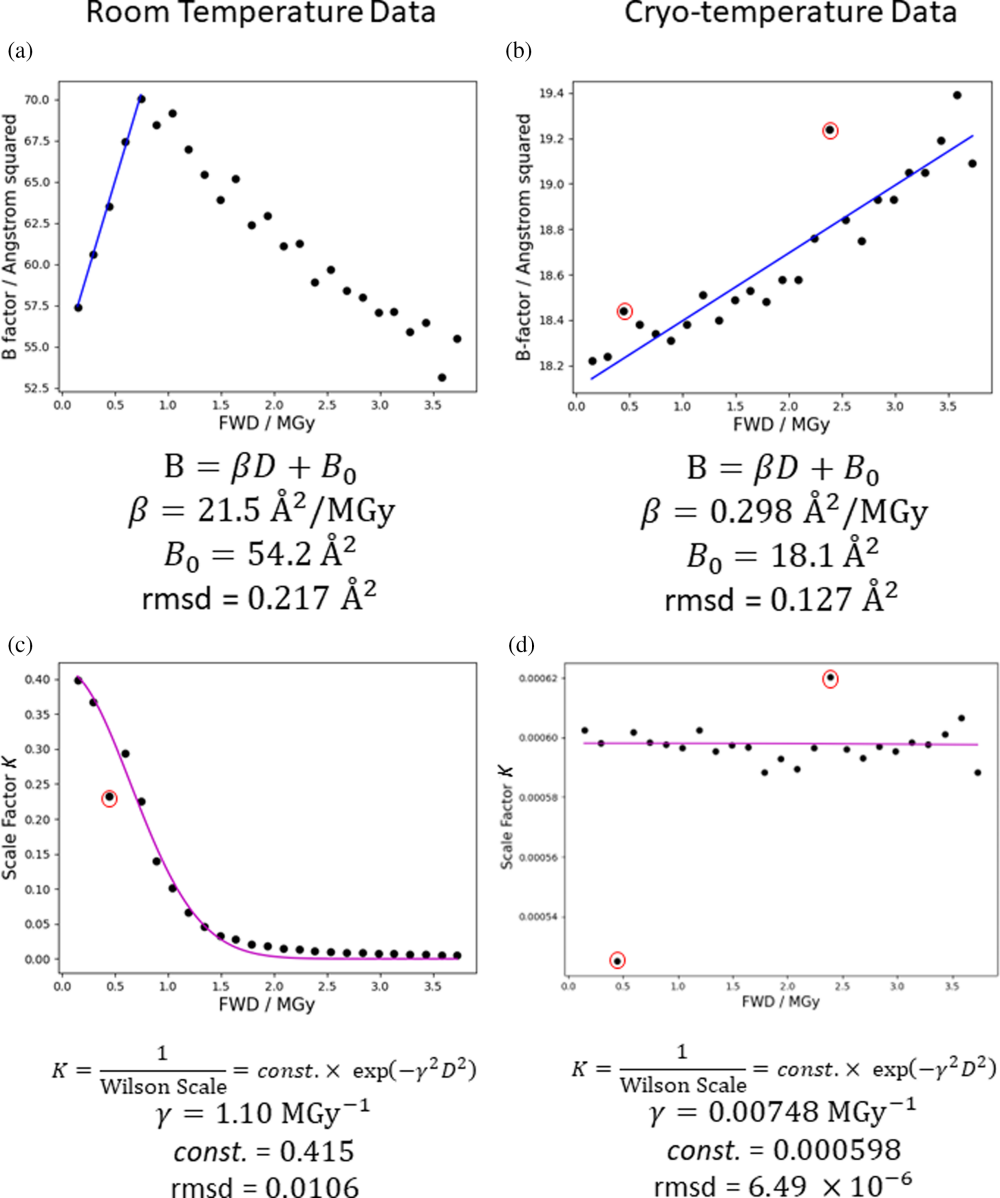
Fitting the IDM proposed by Leal et al. (2013) to (a, c) the room temperature high dose rate and (b, d) cryo‐temperature data from de la Mora et al. (2020). Anomalous data points (indicated by red circles) were excluded during fitting but still shown on the graph: at ≈0.45 MGy for the room temperature data, and ≈0.45 and ≈2.38 MGy for the cryo‐data.

Wilson *B*‐factors and scale factors were calculated for each 2 ms exposure dataset by AIMLESS (Evans & Murshudov, 2013). A maximum resolution limit of 1.71 Å was always specified, because for the data at the longest exposure times higher resolutions than this showed some noise in their Wilson plots, and it was important to ensure the same region of reciprocal space was followed over all exposure times. The Wilson *B*‐factor and the scale factor K (the reciprocal of the Wilson scale factor, s) were plotted against the FWD estimated by RADDOSE‐3D. Fitting of the *B*‐factor term of the Leal et al. (2013) model was performed by least‐squares regression over the region where the *B*‐factor plot is still linear (FWD ≲0.8 MGy), as shown in Figure 4. The scale factor term, K, was fitted by least‐squares regression over the whole data range as indicated in Figure 4. The estimated parameter values agree well (within a factor of two) with previously reported values for chicken egg white lysozyme (HEWL) (Leal et al., 2013). The cryo‐temperature dataset from the same study was also analysed for the same range of FWD values and similarly gave parameter values broadly consistent with previous studies (Leal et al., 2013).

We fit the model to the FWD rather than to another dose metric (e.g., total dose) because FWD accounts for the fact that the dose absorbed by the regions of the crystal that are experiencing more intense regions of the beam has proportionally more impact on the relative intensity and should be given a greater weight. More precisely, fitting an IDM against the FWD is equivalent to making the following approximation:

(5) $$\begin{array}{l} \frac{\int_{t_{i-1}}^{t_{i}}\int_{\text{crystal}}\eta \left(D\left(\vec{x},t\right),h\right)F\left(\vec{x},t\right)d\vec{x}\mathrm{dt}}{\int_{t_{i-1}}^{t_{i}}\int_{\text{crystal}}F\left(\vec{x},t\right)d\vec{x}\mathrm{dt}}\\ \;\approx \approx \eta \left(\frac{\int_{t_{i-1}}^{t_{i}}\int_{\text{crystal}}D\left(\vec{x},t\right)F\left(\vec{x},t\right)d\vec{x}\mathrm{dt}}{\int_{t_{i-1}}^{t_{i}}\int_{\text{crystal}}F\left(\vec{x},t\right)d\vec{x}\mathrm{dt}},h\right) \end{array}$$

where $\eta \left(D,h\right)$ is the relative diffraction efficiency for each region of the crystal as defined as a function of D and h by Equation (3) (noting that η=1 at zero dose), and all other terms are as defined in Equation (1). The close agreement between the DDWD calculated as described in Section 2.1.2 using the model fitted in this way, and the disulphide difference density (Figure 2) suggests that obtaining model parameters through fitting using the FWD is a useful strategy. This close agreement also shows that the dose‐dependent scale factor term K is not a correction factor that should be included only on the right‐hand side of Equation (5) to account for the non‐uniform distribution of $F\left(\vec{x},t\right)$, but instead measures an underlying RD process and is thus a required term within $\eta \left(D,h\right)$ on both sides of Equation (5). This conclusion is further supported by datasets where whole crystals are uniformly irradiated, such as reported in Brooks‐Bartlett (2016), where the scale factors and *B*‐factors display the same trends as shown in Figure 4. The fact that we are fitting the model to apply to each region of the crystal by using reflection intensity data from the whole crystal plotted against the FWD also justifies why we should fit the model only to the initial linear region of the Wilson *B*‐factor plot (Figure 4a). As we will discuss further in Section 2.2.4, beyond the initial linear region the average *B*‐factor of a whole crystal decreases because it is calculated from only measurable diffraction. This results in an equivalent effect to that explaining the decrease in DDWD at high doses (see comparison between Wilson *B*‐factor and DDWD in Figure 2).

#### 2.2.3 Interpretations of the adapted scaling model

For the purposes of DDWD calculation, the underlying meaning of the terms in the Leal et al. (2013) model is not important, as it is only used to provide an accurate prediction of the diffracted flux exiting each region of the crystal. However, to motivate further work towards an IDM that has predictable parameters, the physical basis of the Leal et al. (2013) model will now be discussed.

The Leal et al. (2013) model can be interpreted in kinetic terms, inspired by previous kinetic models (Hendrickson, 1976; Sygusch & Allaire, 1988), as explained when the model was originally proposed (Leal et al., 2013). Assume that the crystal contains two fractions of atoms (i.e., scattering sources), first a fraction that contributes to Bragg diffraction, P_Bragg_, and a fraction that no longer contributes to diffraction, P_None_. The atoms within the Bragg fraction will accumulate small and numerous displacements in proportion to the absorbed dose. As discussed in Section 2.2.1, under a central limit theorem, we derive a linear increase in the *B*‐factors of these atoms with dose, from an initial value, such that:

(6) $${\mathrm{RI}}_{\text{Bragg}}=P_{\text{Bragg}}\exp\left(-\frac{1}{2}\left(B_{0}+\mathrm{\beta D}\right)h^{2}\right)$$

where RI_Bragg_ is the contribution of $P_{\text{Bragg}}$ to the relative intensity.

However, it is also necessary to account for the conversion of P_Bragg_ to the fraction of atoms that no longer contribute to Bragg diffraction due to RD (P_None_). This may be related to the progression of defects in the crystal lattice on large scales that effectively reduce the number of unit cells exposed to the beam, as proposed by Leal et al. (2013) and discussed further in Section 2.2.4.

Whatever the cause, if we assume this “Bragg to None” conversion occurs at a rate with respect to dose that is directly proportional, by a rate constant $2\gamma^{2}$, to the size of the Bragg fraction and to the dose, D, then we have:

(7) $$\frac{dP_{\text{Bragg}}}{\mathrm{dD}}=-2\gamma^{2}DP_{\text{Bragg}}$$

Solving this equation for P_Bragg_ as a function of dose we find that:

(8) $$P_{\text{Bragg}}=P_{0,\text{Bragg}}\exp\left(-\gamma^{2}D^{2}\right)$$

where P_0, Bragg_ is the value of P_Bragg_ at zero dose.

Substituting Equations (8) into (6), and assuming that the only contribution to the total relative intensity is due to the fraction P_Bragg_ (and thus P_0, Bragg_ = 1), gives the same form as the Leal et al. model:

(9) $$M\left(D,h\right)=\exp\left(-\gamma^{2}D^{2}\right)\exp\left(-\frac{1}{2}\left(B_{0}+\mathrm{\beta D}\right)h^{2}\right)$$

The parameter γ could in principle be predicted from the rate constants of the various physical and chemical processes that bring about the “Bragg to None” conversion.

Another hypothesis for the origin of a dose‐dependent term besides the *B*‐factor term is that RD‐induced changes to unit cell size and mosaicity occur at a greater rate than the increase in overall *B*‐factor with dose (proposed by Warkentin et al. (2017) in the context of lag phases). Depending on the crystal orientation relative to the beam, for a subset of diffraction images, this may cause a few reflections to broaden or migrate into the measured region of reciprocal space and thus their measured intensity will increase. However, the total diffracted intensity should be calculated over a large region of reciprocal space sampling thousands of reflections and so the impact of a few reflections on the intensity statistics should be small.

#### 2.2.4 *Physical limits for* B*‐factors and implications for macroscopic crystal stability*

The Leal et al. model predicts that the average *B*‐factor increases indefinitely as dose increases, which is physically impossible within the constraints of a crystal lattice. According to this model, with parameters fitted as in Figure 4, within the dose range of the room temperature dataset the average *B*‐factor calculated according to $B_{0}+\mathrm{\beta D}$ increases to as high as 129 Å^2^ at 3.5 MGy. We might compare this to the *B*‐factor of a bulk solvent model where no solvent mask is specified: usually $B_{\mathrm{sol}}\approx$ 125–200 Å^2^ (Weichenberger et al., 2015) (although this is not formally a *B*‐factor of the solvent atoms it does quantify, for the whole unit cell, the contribution of the solvent in reciprocal space). Eventually, the contribution to diffraction of an atom with high *B*‐factor becomes negligible relative to the sensitivity of the detector. However, probably before it reaches these large values, the average *B*‐factor will be so high that it is physically unreasonable for describing atoms constrained in an ordered crystalline lattice. This is because the integrity of the crystal lattice at the mesoscopic/macroscopic scale emerges from the structural integrity of the macromolecules within the lattice and the contacts between them. It is therefore sensitive to microscopic atomic displacements that result from ionisation events and subsequent radiochemistry; large atomic displacements should disrupt the integrity of the crystal. IDMs should have a term to account for this, and this may be the origin of the scale factor term $K=\exp\left(-\gamma^{2}D^{2}\right)$ in the Leal et al. (2013) model.

As described in Section 2.2.1, a linear increase of *B*‐factor as dose increases is expected only if certain conditions are met: that radiation‐induced atomic displacements are randomly distributed, small but numerous, and accumulate in proportion to the dose (Borek et al., 2013). Furthermore, the definition of the *B*‐factor assumes a crystal with intact unit cells. Most microscopic phenomena such as bond breakage are likely to satisfy these conditions. By definition, microscopic phenomena involve small perturbations, and since the perturbations are small and localised they are more likely to be randomly distributed from the perspective of a whole crystal. Conversely, mesoscopic/macroscopic structural breakdown within the crystal may involve larger displacements (on the scale of whole unit cells) which may be concerted (i.e., not totally random). Thus, crystal lattice breakdown may be a RD process that is not well modelled by a linear average *B*‐factor increase. Again this is consistent with the scale factor term K in the model of Leal et al. being due to the contribution of crystal structural breakdown, as suggested when the model was originally proposed (Leal et al., 2013). Because breakdown of the macroscopic crystal lattice causes atomic displacements, we might expect it to contribute to changes in the apparent average *B*‐factor. However, crystal breakdown should instead reduce the number of intact unit cells because the apparent *B*‐factor loses its physical meaning if we consider scattering from regions of the sample that are no longer crystalline.

More comprehensively than an average *B*‐factor, we might consider the dose‐dependent shift to the full distribution of atomic *B*‐factors, $p_{B}\left(D\right)$. Global damage is then this whole distribution shifting to higher values, whereas specific damage is represented by specific atoms that have *B*‐factors that shift by relatively more than other atoms as dose increases (Gerstel et al., 2015). To formulate an IDM in the form $M\left(D,h\right)$ we need to consider the distribution $p_{B}$ that would be calculated for a small region of the crystal if we had knowledge of the true atomic positions and motions of all atoms in each unit cell within this region (if we fit the IDM to the FWD, the relevant $p_{B}$ is for the whole crystal and has the contribution of unit cells weighted by their incident fluence). Importantly, as dose increases these distributions will become significantly different to the diffraction‐decay weighted atomic *B*‐factor distribution that would be calculated from processing a diffraction pattern all the way to a refined structure, to which only measurable diffraction contributes.

**FIGURE 5:**
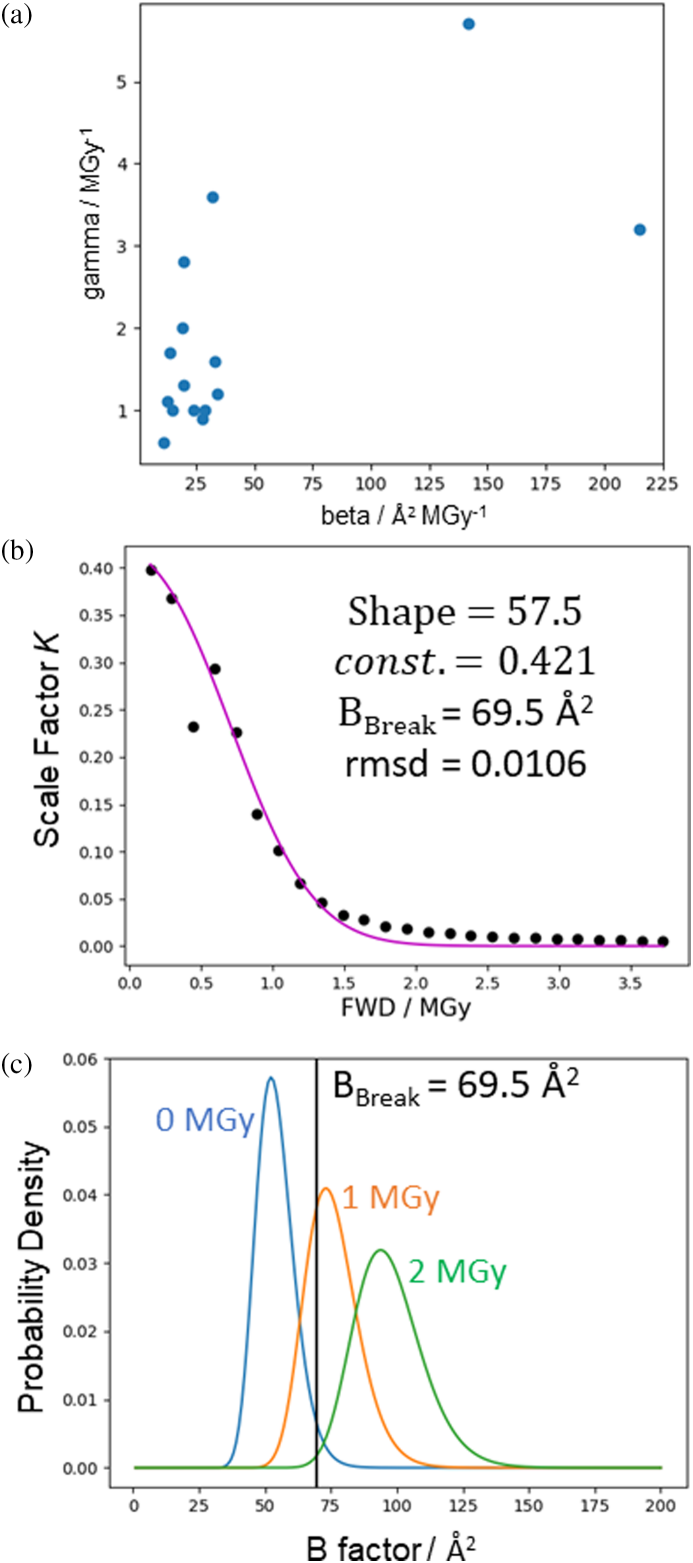
Simplified model for the scale factor *K* in terms of dose‐dependent change to the atomic *B*‐factor distribution, fitted to the room temperature high‐dose rate dataset from (de la Mora et al., 2020). (a) Correlation between β and γ values reported by Leal et al. (2013) (Spearman's rho = 0.474, Pearson's *r* = 0.656) which is consistent with the corresponding *B*‐factor and scale factor terms modelling the same underlying mechanism. (b) Predicted curve and parameter values for the model fitted against the experimental dose‐dependence of the scale factor K. The shape, a, is a parameter of the model used for the atomic *B*‐factor distribution (see Supplementary section 1.2.3 for details), and *const*. is a proportionality constant. (c) For the parameter values from (a), how the modelled atomic *B*‐factor distribution would change with dose. The area under the curve to the left of $B_{\text{Break}}$ is proportional to the scale factor K.

$p_{B}$ is defined in terms of individual atoms (and assuming a crystal with intact unit cells). By the same logic as applied in the preceding discussion of the average *B*‐factor, shifts in $p_{B}$ are most easily rationalised in terms of microscopic phenomena, for example bond breakage is associated with increased *B*‐factors of the atoms involved in the bond. We would like to model how RD at this microscopic level might propagate to mesoscopic/macroscopic defects in the crystal. For the model of Leal et al., the parameters β and γ are correlated (as shown in Figure 5), which is consistent with the scale factor and *B*‐factor terms of this model both ultimately arising from the same or related phenomena at the microscopic level.

Figure 5 shows the simplest possible model for how a dose‐dependent shift to $p_{B}$ could result not only in an increasing average *B*‐factor but also a term like the scale‐factor K. For the purposes of illustration, $p_{B}$ is taken to be an inverse gamma function with a mean that increases according to the *B*‐model (i.e., $B_{0}+\mathrm{\beta D}$, see Supplementary materials, section 1.2.3 for details). To produce a term with behaviour similar to the scale factor term K, we make two further assumptions. First, the extent of mesoscopic/macroscopic lattice defects is proportional to the fraction of atoms with *B*‐factors above a certain threshold, $B_{\text{Break}}$, because we assume the crystal lattice is robust to displacements of individual atoms only up to a certain limit. Second, the decay in diffraction intensity which cannot be explained by the linear average *B*‐factor increase is directly proportional to the extent of these defects, so the defects proportionally reduce the effective number of exposed unit cells. Figure 5 shows the fitting of this model to the variation of K with dose and shows how $p_{B}$ varies with dose according to the fitted model. Inspection of Figure 5c suggests the exact distribution used to model $p_{B}$ should not have a huge impact on the predictions of this model so long as it is approximately bell‐shaped and the whole distribution shifts past $B_{\text{Break}}$ to higher values as dose increases.

The best fit for $B_{\text{Break}}$ approximately matches the value at which the observed linear relationship between Wilson *B*‐factor and dose breaks down. This is expected because in this model the combination of the linear relationship ($B=B_{0}+\mathrm{\beta D}$) and the parameter $B_{\text{Break}}$ determines the doses at which the scale factor term goes through a steep decrease, which has a large effect on the relative intensity and thus the measured value of diffraction‐weighted quantities (see end of this section for further discussion). This value of $B_{\text{Break}}$ represents a mean square atomic displacement of $\left\langle u^{2}\right\rangle =\frac{B}{\left(8\pi^{2}\right)}=0.88\;{Å}^{2}$. If the many assumptions underlying this simple model hold, we can interpret this as a maximum average square atomic displacement that can be tolerated by the intact crystal lattice for this particular sample.

In this formulation, we have considered the *B*‐factor distribution of all atoms. However, it is necessary to give greater weight to the subset of atoms that are relatively more important for the structural integrity of the lattice (e.g., atoms at crystal contact sites). This has no effect on the model predictions if the atomic *B*‐factor distribution of these atoms mirrors the *B*‐factor distribution of all atoms. However, there will evidently be sample‐dependent exceptions to this. A particularly striking example is crystals of oligomeric dodecin, where the decarboxylation of Glu57 probably causes a destabilisation of oligomers (and hence crystalline order) leading to global RD much faster than expected (Bieger et al., 2003; Murray et al., 2005). Further limitations of this treatment include the fact that by considering only atoms with *B*‐factors it neglects any effect of radiation‐induced changes to bulk solvent on the crystal lattice (e.g., an internal pressure due to gas generation) unless these have a comparable effect on the atomic *B*‐factor distribution. Furthermore, it does not directly model phenomena where causation occurs at the macroscopic level of the crystal lattice (e.g., mechanical forces propagated by the lattice, which might drive cooperativity and spreading of defects) and reduces mesoscopic/macroscopic lattice stability to just a single parameter, $B_{\text{Break}}$. Evidently, our understanding would also be improved by a more accurate model for $p_{B}$ and how $p_{B}$ changes with dose, and a way to quantify the contribution of individual atoms to mesoscopic/macroscopic lattice stability. We hope that future models can improve on the simplistic $B_{\text{Break}}$ model.

The linear increase in the apparent Wilson *B*‐factor with dose breaks down at ≈0.8 MGy (see Figure 2). This is likely because the calculated Wilson *B*‐factor is itself “diffraction decay‐weighted” given it is calculated from the measurable diffraction. The behaviour of the Wilson *B*‐factor approximately matches the behaviour of DDWD (see Figure 2c), which is calculated assuming the model of Leal et al. (2013) holds over the full dose range analysed. The scale factor term, K, has a large effect on the diffracted intensity and the linearity of the Wilson *B*‐factor plot breaks down as K rapidly decreases between 0.5 and 1.0 MGy. If we accept that the decreasing scale factor term represents a structural breakdown of the crystal lattice, beyond FWD ≈0.8 MGy it is unclear whether defining *B*‐factors is even meaningful for the most damaged regions of the “crystal” which receive a local dose well in excess of 1 MGy and have probably essentially become amorphous. The fact that calculating Wilson *B*‐factor from the whole crystal is at all possible is due to diffraction from the weakly illuminated regions of the crystal that have only received a relatively low dose.

### 2.3 IDMs and DDWD: Outlook

In this work we have shown how, by incorporating the IDM by Leal et al. (2013) into RADDOSE‐3D, the code can be used to calculate the DDWD. This DDWD can be used to understand the extent of RD in an electron density map, exemplified by its correlation with the difference density of a disulphide bond (de la Mora et al., 2020). The parameters of the IDM, as estimated by fitting to global intensity statistics (Wilson *B*‐factor and scale factor), could be used in the DDWD calculation (which applies the IDM to each region of the crystal to determine the diffracted flux scattered from that region).

However, it would be even more advantageous to be able to predict the parameter values of the IDM in advance of the experiment, without the need to fit them to each specific diffraction dataset. This work has examined a range of possible frameworks for describing the physical and chemical changes to the crystal as dose increases, which give rise to the form and parameter values of the IDM. These include conversion between crystal fractions according to kinetic rate equations, and dose‐dependent increases to atomic *B*‐factors (the average *B*‐factor or more exactly a shift to the entire distribution of atomic *B*‐factors). There is sample‐dependent influence on these processes, as evidenced by the large table of parameter values in Leal et al. (2013), and also suggested by the range of h exponent values in Atakisi et al. (2019), which will need to be understood before a generally predictive IDM can be formulated. Sample sensitivity to global damage will vary due to a range of factors relating to the unit cell contents and the structural integrity of the crystal lattice. First, analogous to how different proteins have different rates of specific damage with respect to dose due to their exact structure (e.g., disulphide bond breakage depending on the angle made with a carbonyl oxygen (Bhattacharyya et al., 2020), and staple‐group disulphides having greater susceptibility (Gerstel et al., 2015)), different proteins may have slightly different susceptibility to breakage of bonds between their light elements, even if the large number of such bonds in a given protein molecule should average out most variation. Second, the complex chemistry known to occur during RD (including the formation and propagation of radicals (Owen et al., 2012) and of hydrogen gas (Meents et al., 2010)) suggests that slightly different amino acid compositions and environments within the crystal may have some influence on radiation sensitivity. Thirdly, differences in crystal packing may impose different constraints on unit cell expansion and increased mosaicity which may contribute to the intensity decay, in addition to more direct effects such as those observed for the highly radiation‐sensitive crystals of oligomeric dodecin discussed in Section 2.2.4 (Bieger et al., 2003; Murray et al., 2005). These sample‐dependent influences could be dissected experimentally by using an independent technique to monitor the damage state of the crystal as data collection progresses (e.g., spectroscopy (Fernando et al., 2021) or X‐ray topography (Suzuki et al., 2022)). Also useful would be a systematic comparison of intensity decay curves for a broader range of proteins than the standard model proteins (HEWL, insulin, thaumatin). In particular, it may be desirable to study different crystal forms of the same protein (due to alternative functional conformations or induced, for example, by changing the crystal temperature (Weik et al., 2001)), or compare sequence variants that have different melting temperatures or are functional at different temperatures *in vivo*. We speculate that strategies optimised for extracting predictive power despite a large parameter space will be optimal for solving this problem, as would a more detailed understanding of the dynamics of radiolysis and subsequent chemistry.

Improved predictive power of IDMs would have implications for crystallographic data analysis. Scaling programs, such as AIMLESS (Evans, 2006; Evans & Murshudov, 2013), XDS (Kabsch, 2010), HKL3000 (Minor et al., 2022), and DIALS (Beilsten‐Edmands et al., 2020), must account for intensity decay due to RD as part of placing reflections on a consistent scale through application of scale, decay and absorption terms, leveraging multiplicity within the dataset. The decay factor is usually an overall *B*‐factor correction analogous to that described in the IDMs in Table 1. The scale factor also contributes to correction for the effects of global RD, where these effects cannot solely be taken into account by the *B*‐factor correction (i.e., the scale factor is dose‐dependent as in the IDM by Leal et al. (2013)) but in the context of scaling it is also important as a more general correction, for example, for changes to the illumination volume. Because scaling must only be consistent within a dataset, these programs use an experimental coordinate such as frame number, rotation angle, or exposure time as a proxy for dose, rather than the absolute dose estimated through consideration of the physics of X‐rays interacting with the crystal, for example, by RADDOSE‐3D. Some scaling programs provide an option for zero‐dose extrapolation for individual reflections using linear, polynomial, or exponential functions (Borek et al., 2007; Diederichs et al., 2003; Kabsch, 2010). However, these attempts are complicated by the fact that individual reflections do not all follow the average trend described by IDMs (e.g., some reflection intensities actually increase, as noted by Blake and Phillips in 1962 [Blake & Phillips, 1962]), and by the need to handle negative values of experimentally measured intensities and weak intensities. Attempts to improve zero dose extrapolation have included using the Wilson intensity distribution as a Bayesian prior or encoding IDMs into the process function of a Hidden Markov model describing the evolution of the crystal with increasing dose (Brooks‐Bartlett, 2016). Additional corrections may also be applied to account for anisotropy induced by RD.

It has generally been assumed that the intensity decay due to global damage is largely separable from the effects on the intensities due to specific damage processes. Specific damage requires that damage events occur at the same atomic position in all unit cells of the crystal. Hence, it is usually detectable in real space only for radiation‐sensitive sites that damage at a greater rate (with respect to dose) than the majority of positions within the unit cell. Recent progress in quantifying specific damage includes using singular value decomposition to model it as a component distinct from global damage in reciprocal space (Borek et al., 2013), its separation into individual components in real space through independent component analysis (Borek et al., 2018), and analysis of atomic *B*‐factors in real space through the *B*_Damage_ metric (Gerstel et al., 2015), and the related metric *B*_net_ that can be calculated for a whole structure and enable comparison between structures (Shelley & Garman, 2022). Conversely, inherent in the derivation of an isotropic global scaling *B*‐factor (i.e., that encoded in the “B‐model”) through a central limit theorem is the fact that damage events occur randomly according to a uniform distribution across the atomic positions in the unit cell. Conceptually, the observed distribution of *B*_Damage_ values (Gerstel et al., 2015) could result from either a homogeneous distribution of absorbed dose within an average unit cell but different activation energies for damage at different positions, or an inhomogeneous distribution of absorbed dose within an average unit cell, or probably a combination of the two possibilities. The input energy required for atomic displacements and bond breakage is a function of temperature, as is the distribution of absorbed dose due to molecular motion, secondary damage, and mobile ions/radicals. The DDWD estimated by RADDOSE‐3D is proportional to the dose per average unit cell represented by an electron density map, and so would be the appropriate dose metric for comparison to atomic *B*‐factor distributions of refined structures. Therefore, we expect the implementation of DDWD in RADDOSE‐3D will be a further useful tool for scientists wanting to characterise the extent of RD in their structures.

## 3 RADDOSE‐ED

Although X‐ray crystallography has enabled us to determine the structures of a wide variety of molecules from small molecules to comparatively large macromolecular complexes (Shi, 2014), the technique is reliant on the successful production of large, well‐diffracting crystals. To successfully obtain a structure from a single protein crystal, Holton & Frankel (2010) predicted that a spherical crystal must be at least 1.2 μm in diameter, or potentially 0.34 μm if there is significant photoelectron escape. This theoretical limit has not yet been reached, with the smallest crystals successfully used for structure determination having scattering powers ≈15× larger than a 1.2 μm sized crystal. This size limit can be somewhat reduced by using multiple crystals, as in serial synchrotron crystallography (SSX) (Gati et al., 2014; Stellato et al., 2014), and reduced even further if RD can be outrun, as is the case for SFX at XFELs (Chapman et al., 2014; Nass, 2019). Nonetheless, although possible using SFX (Colletier et al., 2016), sub‐micron‐sized crystals still present major difficulties for successful structure determination using X‐rays.

Electrons are theoretically much more suited than X‐rays for imaging thin samples. As calculated by Henderson (1995) by comparing scattering cross sections, electrons offer approximately three orders of magnitude more signal per unit radiation dose for very thin specimens. As a result of this, there is a long history of structure determination using electron crystallography. Traditionally, this has involved using 2D crystals that consist of just a single monolayer of molecules. The first protein structure to be determined by 2D crystallography was of purple membrane (Henderson & Unwin, 1975) using a combination of both diffraction patterns and images. More recently, the methodology has been extended further to 3D crystals, in a technique often called MicroED (Shi et al., 2013). This has proved useful in determining structures from crystals that do not grow large enough for successful structure determination using X‐rays (Clabbers et al., 2022), and structures from single crystals more than 2 orders of magnitude smaller than that achieved using MX have been solved (Rodriguez et al., 2015).

On the other hand, the much higher scattering cross sections of electrons compared to X‐rays make it exceedingly difficult to determine structures from samples more than just a few hundred nanometres thick using beam energies commonly available in modern transmission electron microscopes. Experimental studies suggest that solving structures from crystals thicker than 2 inelastic scattering mean free path lengths (MFP lengths, ≈600 nm for 300 keV electrons) is extremely difficult (Martynowycz et al., 2021). As a result, crystals too large for MicroED are often thinned using focused ion beam (FIB) milling (Duyvesteyn et al., 2018; Martynowycz et al., 2019).

The fundamental cause of the limitation in crystal size for both electrons and X‐rays is RD since it limits the amount of signal we can achieve before the molecules are destroyed. RD studies in X‐ray crystallography have benefited from using dose as a metric against which to monitor its manifestations, enabling us to compare its progression between datasets from a variety of samples and under different data collection conditions. This has accelerated our understanding of RD to biological macromolecules from ionising radiation as well as allowing the optimisation of data collection strategies to improve the chance of successful structure determination. In electron crystallography, “dose” has typically been reported in terms of fluence (e^−^/Å^2^), but this fails to account for other factors that determine the dose such as primary beam energy and sample composition. Since this makes inter‐comparisons and thus finding optimisation strategies challenging, some efforts have been made to convert e^−^/Å^2^ to gray (Baker & Rubinstein, 2010; Egerton, 2021), but this change has so far not been widely adopted.

RADDOSE‐3D (Bury et al., 2018; Zeldin, Gerstel, & Garman, 2013) has been used by many as a simple tool to estimate dose for X‐ray crystallography experiments, and more recently SAXS (Brooks‐Bartlett et al., 2017) and SFX experiments (Dickerson et al., 2020). However, it has so far been limited only to calculations for incident X‐rays, not having been written for other projectiles. We have now extended RADDOSE‐3D to calculate doses for electron irradiation; specifically electron crystallography experiments in the subprogram RADDOSE‐ED. We demonstrate that RADDOSE‐ED can be used to convert fluence to dose for electron crystallography experiments and that for a given amount of absorbed dose, the extent of RD is comparable to that in X‐ray crystallography. Moreover, by calculating an information coefficient (Peet et al., 2019), defined as the signal intensity per MGy of absorbed dose, we demonstrate how RADDOSE‐ED can be used to optimise the beam energy for a given specimen thickness.

### 3.1 Methods

RADDOSE‐ED calculates electron stopping powers to estimate the energy absorbed by the sample, and hence estimate the dose. It also calculates elastic and inelastic scattering cross sections, allowing estimation of the information coefficient. RADDOSE‐ED can be run using a standard RADDOSE‐3D input file, but with an additional flag “Subprogram EMED” in the crystal block. The electron fluence is also given in e^−^/Å^2^ instead of specifying an X‐ray flux in photons/s.

#### 3.1.1 Calculation of dose for incident electrons

To calculate the dose in Gy, we must calculate the mass of the exposed volume, as well as the energy absorbed by the sample. The mass is calculated as the sum of the masses of all atoms in the exposed area. The absorbed energy is calculated using the electronic stopping power for electrons. The total electronic stopping power is the sum of two stopping power components, collision stopping power, $S_{\mathrm{col}}$, and radiative stopping power, $S_{\mathrm{rad}}$. The collision stopping power is the average energy loss per unit path length as a result of Coulomb collisions with bound atomic electrons, resulting in ionisations and excitations (Brice, 1985). The radiative stopping power is the average energy loss per unit path length due to the emission of Bremsstrahlung in the electric field of the atomic nucleus and atomic electrons.

The collision stopping power for an atom, $S_{\mathrm{col}}$, is calculated in a similar way as described in RADDOSE‐XFEL (Dickerson et al., 2020) and is as follows: (Bethe, 1930; Bethe, 1932).

(10) $$S_{\mathrm{col}}=\rho \frac{2\pi N_{a}r_{e}{\mathrm{mc}}^{2}}{\beta^{2}}\frac{Z}{A}\left(F\left(\beta \right)-2\ln I-\delta \right)$$

(11) $$F\left(\beta \right)=\begin{array}{l} \ln\left(\frac{{\mathrm{mc}}^{2}E_{e}\beta^{2}}{2\left(1-\beta^{2}\right)}\right)-\left(2\sqrt{1-\beta^{2}}-1+\beta^{2}\right)\ln2+1\\ -\;\beta^{2}+\frac{1}{8}\left(1-\sqrt{1-\beta^{2}}\right) \end{array}$$

(12) $$\beta =\sqrt{1-\frac{1}{\gamma^{2}}}$$

(13) $$\gamma =1+\frac{E_{e}}{{\mathrm{mc}}^{2}}$$

where ρ is the crystal density, $N_{a}$ is Avogadro's number, $r_{e}$ is the classical electron radius, m is the electron rest mass, c is the velocity of light, Z is the atomic number, A is the atomic mass, $E_{e}$ is the incident electron kinetic energy, I is the mean excitation potential, and δ is the density effect correction. I is an experimentally determined parameter that is tabulated in ICRU report 37 (ICRU, 1984), and these values are multiplied by the constant 1.13 to modify them from the gas phase to the liquid/solid phase (ICRU, 1984).

The density effect correction is applied since the passage of electrons through a medium polarises atoms and this polarisation in turn decreases the electromagnetic field acting on the particle, reducing the stopping power. The size of δ increases with the density of the material and the kinetic energy of the electron. This was calculated according to the fits provided by Sternheimer et al. (Sternheimer, 1952; Sternheimer et al., 1984) as follows:

(14) $$\delta =\left(\begin{array}{ll} 4.06x+C+a{\left(x_{1}-x\right)}^{b}, & {\mathrm{if}\;x}_{0}<x<x_{1}.\\ 4.06x+C, & x>x_{1}. \end{array}\right.$$

(15) $$x=\log_{10}\left(\gamma \beta \right)$$

where $x_{0}$ is the value of x below which δ=0, $x_{1}$ is the value above which the relation between x and δ can be considered linear, and a, b, and C are constants dependent on the element (Sternheimer, 1952).

The collision stopping power for the entire sample is obtained using the Bragg additivity rule, stating that the collision stopping power for a compound is the weighted sum of the atomic constituents. This is equivalent to replacing the Z/A term with:

(16) $$\left\langle Z/A\right\rangle =\sum_{j}\omega_{j}\left(\frac{Z_{j}}{A_{j}}\right)$$

where j denotes the j'th atomic constituent and $\omega_{j}$ is the fraction of the total molecular weight in the unit cell that the j'th atom contributes. The mean excitation energy and density effect correction are also modified accordingly:

(17) $$\ln I=\frac{1}{\left\langle Z/A\right\rangle }\left[\sum_{j}\omega_{j}\left(\frac{Z_{j}}{A_{j}}\right)\ln I_{j}\right]$$

(18) $$\delta =\frac{1}{\left\langle Z/A\right\rangle }\left[\sum_{j}\omega_{j}\left(\frac{Z_{j}}{A_{j}}\right)\delta_{j}\right]$$

For the incident electron energy ($E_{e}$) range of interest, the radiative stopping power constitutes a much smaller contribution to the total stopping power than the collision stopping power. For instance, the collision stopping power is 99.8% of the total stopping power of liquid water at 300 keV, and 98.6% at 2000 keV (ICRU, 1984). Since this contribution is so small, we used an estimate of the radiative stopping power, $S_{\mathrm{rad}}$ as follows (Hussein et al., 2023):

(19) $$S_{\mathrm{rad}}=S_{\mathrm{col}}\left(\frac{E_{e}\left\langle Z\right\rangle }{800}\right)$$

where $\left\langle Z\right\rangle$ is the mean Z by mass. The total stopping power is then the sum of the radiative and collision stopping powers.

#### 3.1.2 Scattering cross sections

For energies $E_{e}\leq 300$ keV, both the inelastic and elastic scattering cross sections for each element are calculated in the same way as in RADDOSE‐XFEL (Dickerson et al., 2020), which calculates the stopping power of photoelectrons of energies on the order of that of the incident X‐rays. The elastic scattering cross sections are taken from tabulated values (Jablonski et al., 2016). The inelastic scattering cross sections are calculated using the generalized oscillator strength model for outer shell collisions (Sempau et al., 2001), and a combination of the plane‐wave Born approximation and distorted‐wave Born approximation for inner shell collisions (Bote et al., 2009). For energies greater than 300 keV, the inelastic scattering cross sections are also calculated similarly. For elastic scattering cross sections, $\sigma_{\mathrm{el}}$, at these energies, a simple formula that matches well with partial wave computations is used (Langmore & Smith, 1992).

(20) $$\sigma_{\mathrm{el}}=\frac{Z^{1.5}}{\beta^{2}}\left(1-\frac{0.26Z}{137\beta }\right)$$

To calculate both inelastic and elastic scattering cross sections for the entire sample, the cross sections are summed for each atom present in the exposed volume and then converted to a MFP. Poisson statistics are then used to determine the number of elastic and inelastic scattering events, which is appropriate since scattering events are independent.

### 3.2 Results

#### 3.2.1 Parameters important for dose calculation

**FIGURE 6:**
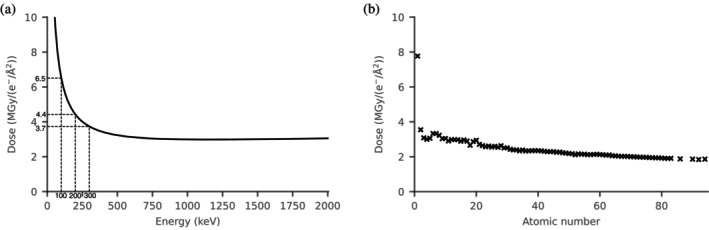
The RADDOSE‐ED estimated dose per e^−^/Å^2^ for a 200 nm cubic crystal of pure low‐density amorphous ice as (a) a function of incident beam energy and (b) atomic composition. The estimated doses are 6.5 MGy/(e^−^/Å^2^) at 100 keV, 4.4 MGy/(e^−^/Å^2^) at 200 keV, and 3.7 MGy/(e^−^/Å^2^) at 300 keV.

We have investigated which parameters are important for accurately estimating the dose in electron crystallography experiments. Doses were estimated in RADDOSE‐ED for a 200 nm cubic crystal of pure low‐density amorphous ice, and the incident beam energy was varied between 10 and 2000 keV in 10 keV steps (Figure 6a). Doses initially steeply drop with increasing beam energy due to a decrease in collision stopping power, before beginning to rise very gradually above 1160 keV as a result of the increasing radiative stopping power.

As well as varying the beam energy, the effect of atomic composition on dose was tested. We used a 200 nm crystal containing 500 of the same atom, ranging in atomic number from 1 to 83, and we also made estimations for radon, thorium, uranium, and plutonium samples. The doses generally decrease with increasing atomic number (Figure 6b), which agrees well with those calculated by Egerton (2021).

#### 3.2.2 Comparison to MX

To determine if RD progresses similarly at cryo‐temperatures in MicroED as it does in X‐ray crystallography at 100 K, the absorbed dose of a MicroED dataset for proteinase K (Hattne et al., 2018) was calculated with RADDOSE‐ED (Table 2). The reduction in the intensity of the diffraction pattern is shown in Table 2 and compares well with values from X‐ray crystallography (e.g., for lysozyme at a temperature of 100 K), a $D_{1/2}$ of 12–14 MGy at 1.6–2.5 Å resolution (Teng & Moffat, 2000) and of 12.5–12.9 MGy at 1.8–35 Å resolution (de la Mora et al., 2011) has been observed. In terms of specific damage in MicroED, the disulphide bonds break before decarboxylation first appears (Hattne et al., 2018), as also observed in X‐ray crystallography (de la Mora et al., 2011).

**TABLE 2:** RADDOSE‐ED calculated dose of a MicroED radiation damage dataset for proteinase K.

Damaging effect	Exposure (e^−^/Å^2^)	Dose (MGy)
$D_{1/2}$ (1.7–21 Å)	2.6	12
S–S bond breakage	0.9	4.0
Decarboxylation first appears	2.0	8.8
All carboxylate groups removed	5.0	22

*Note*: The doses are calculated for the point at which the intensity drops to 50% of that in the first frame ($D_{1/2}$), as well as for where the disulphide bond breakage and decarboxylation of aspartate and glutamate side chains were observed at cryo‐temperature (Hattne et al., 2018).

#### 3.2.3 Information coefficient

As well as estimating the absorbed dose, RADDOSE‐ED also estimates the diffracted intensity per unit dose (“the diffraction efficiency” [Dickerson & Garman, 2019]), which is similar to the information coefficient defined by Peet et al. (2019) but applied to MicroED instead of to single particle cryoEM (SPA). The number of incident electrons that contribute useful signal is assumed to be those that elastically scatter once, and only once, in the sample and do not inelastically scatter. This is because inelastically scattered electrons will broaden the diffraction spots, and multiple elastic scattering (dynamical scattering) breaks the relationship between the Bragg intensities and the single‐scattering values used for structure determination (Glaeser & Downing, 1993; Subramanian et al., 2015).

This number is then divided by the absorbed dose to give the information coefficient. For any given crystal, RADDOSE‐ED will also estimate and output the beam energy that maximises the information coefficient.

**FIGURE 7:**
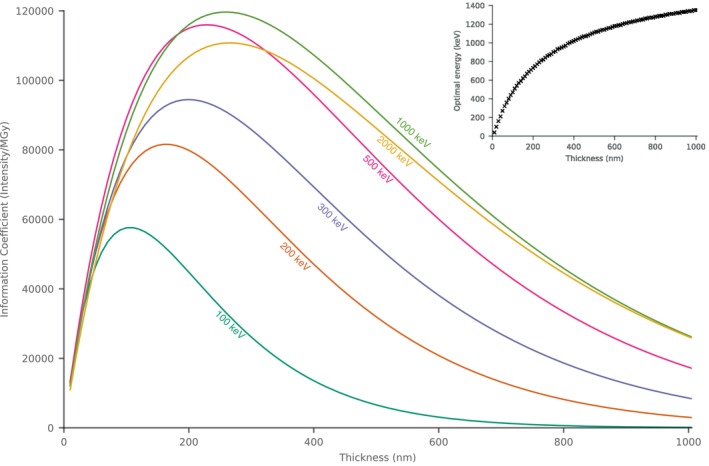
The number of single elastically scattered electrons that have not inelastically scattered per MGy of absorbed dose (information coefficient) versus the thickness of a crystal consisting of pure low‐density amorphous ice. This is plotted for 6 different incident electron energies between 100 and 2000 keV. The optimum incident energy for a given thickness is plotted in the inset. In general, incident electron energies above 300 keV and crystals ≈200 nm thick maximise the information coefficient.

We used RADDOSE‐ED to estimate the information coefficient for crystals of pure low‐density amorphous ice, with thicknesses varying from 10 to 1000 nm in 10 nm steps, and beam energies of 100, 200, 300, 500, 1000, and 2000 keV (Figure 7). RADDOSE‐ED was also used to estimate the beam energy that maximises the information coefficient for a given crystal thickness (inset of Figure 7).

For very thin specimens, such as 2D crystals, lower incident electron energies are more favourable, with an optimal energy of 100 keV. As the specimen gets thicker, the information coefficient increases for all incident energies and peaks between 110 nm (100 keV) and 270 nm (2000 keV). The peak information coefficient increases as the energy increases, with the overall highest being with an 820 keV beam and a 250 nm thick crystal. At energies above this, the peak information coefficient begins to reduce, and there is little or no extra improvement for crystals <1 μm thick. This behaviour is caused by several factors. For thin specimens, lower energies are more beneficial since the ratio of elastic scattering to inelastic scattering is higher (Peet et al., 2019), meaning that there is more signal per unit dose. As specimen thickness increases, the information coefficient increases since the number of scatterers, and hence the diffracted intensity, increases. For thicker specimens, higher incident energies, and thus lower scattering cross sections, are required to minimise losses in signal from inelastic and dynamical scattering. However, since the ratio between elastic and inelastic scattering continues to reduce with increasing energy, there is eventually no improvement for samples thicker than ≈250 nm, leading to an optimum energy of 820 keV.

### 3.3 Discussion: RADDOSE‐ED

We have extended RADDOSE‐3D to include a new subprogram, RADDOSE‐ED, to convert fluences in e^−^/Å^2^ into doses in MGy for electron diffraction experiments. Since account is now taken of primary beam energy and specimen composition, the biggest error in dose is likely to be the fluence measurement itself. Methods to measure this as accurately as possible are discussed by Krause et al. (2021). Unless beam currents are very low, one must be careful when using the counts from a direct electron detector, because of coincidence loss (Li et al., 2013). Measuring the beam current as indicated by the built‐in screen ampere meter can also often be inaccurate. Installing a Faraday cup, or using the drift tube of a spectrometer as a Faraday cup, is often the most accurate method (Krause et al., 2021), and can also be used to properly calibrate the screen ampere meter.

We compared the symptoms of RD (specifically the rates of intensity decay, disulphide bond breakage, and decarboxylation) at cryo‐temperatures in MicroED and X‐ray crystallography, and determined that both global and specific damage events happen at similar doses. Considering that the majority of damage in X‐ray crystallography is from photoelectrons and subsequent electrons produced (O'Neill et al., 2002), this is not surprising.

The information coefficient output by RADDOSE‐ED allows beam energy and crystal thickness to be optimised. For thin crystals, such as 2D crystals, lower energies are predicted to be more optimal. As specimen thickness increases, signal losses due to multiple elastic scattering and inelastic scattering mean that higher energies, where the cross sections are lower, become more favourable. In fact, much higher energies than we are currently using should be used to both maximise the information coefficient and extend the upper limit of crystal thickness that can be productively investigated by MicroED, which has been experimentally measured to be two inelastic MFPs (Martynowycz et al., 2021). However, as the accelerating voltage increases, the size and cost of the microscope also rise, making energies above 300 keV a potentially costly endeavour. Although our results suggest 820 keV is optimum, the improvement above 500 keV becomes relatively modest, making 500 keV perhaps an ideal compromise between cost and maximising the information coefficient.

It is important to note that the information coefficient is not the only metric that will determine the quality of electron diffraction data. The information coefficient only considers signal and not noise; it assumes that electrons that have inelastically scattered, or elastically scattered more than once, are removed. Although this is true for inelastically scattered electrons if an energy filter is used, electrons that elastically scatter more than once (also termed dynamical scattering) are only removed if they scatter beyond the objective aperture. Dynamical scattering is particularly problematic since it breaks the kinematic approximation, meaning that kinematically forbidden reflections will now appear. As a result, diffraction from thick specimens is likely to be worse than predicted by a simple information coefficient, and higher energies thus may be more beneficial than suggested by the results shown in Figure 7. On the other hand, the harmful effects of dynamical scattering can potentially be reduced both experimentally (Clabbers & Abrahams, 2018; Subramanian et al., 2015) and computationally (Clabbers et al., 2019; Klar et al., 2023; Palatinus et al., 2015; Spence & Donatelli, 2021).

For crystals still too thick to be amenable to MicroED, specimens can be thinned by FIB milling to reduce them to the optimum thickness for a given primary beam energy (Duyvesteyn et al., 2018; Martynowycz et al., 2019). However, this will leave a layer of damage, estimated to be 30–60 nm thick for FIB milling ion energies of 30 keV (Berger et al., 2023; Lucas & Grigorieff, 2023; Parkhurst et al., 2023; Tuijtel et al., 2023; Yang et al., 2023). As a result, any FIB milled specimen will have less signal than expected for its thickness and the information coefficients will thus be reduced.

Lastly, although written for electron diffraction, RADDOSE‐ED can in principle be used to convert fluences in SPA or cryogenic electron tomography (cryoET) into doses in MGy. The doses are likely to be a slight overestimate for very thin specimens used for SPA since RADDOSE‐ED does not consider the escape of secondary electrons from the sample. The information coefficient described here does not apply to SPA or cryoET, since in those regimes the experimenter is mostly interested in a specific molecule of a particular size. As specimen thickness increases beyond this size, the signal will only decrease as a result of extra scattering (Dickerson et al., 2022; Russo et al., 2022).

## 4 RADDOSE‐3D GUI

**FIGURE 8:**
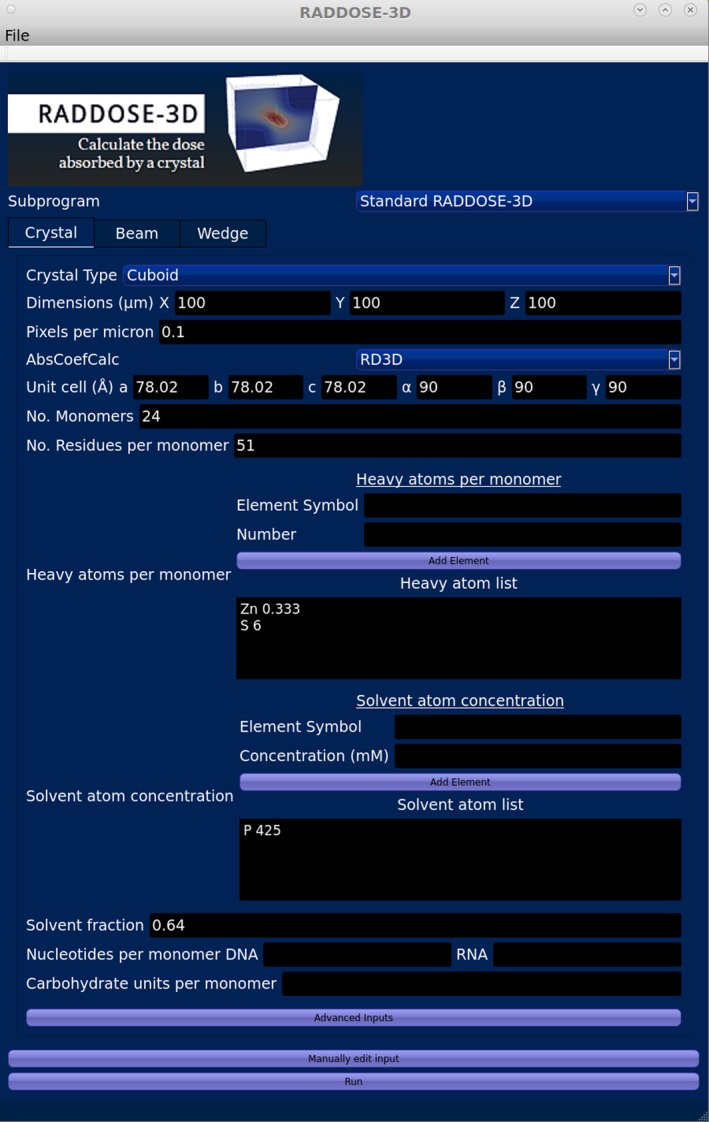
The main window for the RADDOSE‐3D GUI, which shows the inputs for the crystal block of the standard RADDOSE‐3D program.

RADDOSE‐3D has been interfaced at several synchrotron beamlines worldwide and is becoming embedded in assisting in data optimisation strategies. For instance, it has been integrated into Blu‐Ice, used to control data collections at all the MX beamlines at the Stanford Synchrotron Radiation Laboratory, and into GDA at beamline I04 at Diamond Light Source (Masmaliyeva & Murshudov, 2019; McPhillips et al., 2002). To aid experimenters in planning their experiments, we have written an open‐access RADDOSE‐3D GUI in C++ which is suitable for running on both Windows and Linux‐based operating systems (Figure 8). The GUI allows the user to select which RADDOSE utility they wish to run on a drop‐down menu tab at the top of the right hand of the screen which lists: “Standard RADDOSE‐3D”, “XFEL”, “Monte‐Carlo”, and “RADDOSE‐ED.” Once selected, the appropriate data entry boxes are displayed with 3 different tabs for the three blocks of “Crystal”, “Beam”, and “Wedge”. For instance, if a standard RADDOSE‐3D run for MX, SAXS, or SMX is required, the following information about the sample can be entered in the “Crystal” tab: its shape (cuboid, polyhedron, cylindrical spherical), its XYZ dimensions (in μm), and the desired Pixels per Micron (default 0.1: this affects the voxelation and hence the resolution of the calculation). The next drop‐down menu allows the user to specify the absorption coefficient calculation (ACC) appropriate to their experimental modality (MX, SAXS, or SMX): the ACC in‐built in RADDOSE‐3D, EXP (uses a PDB file), SEQUENCE, SAXS, SAXSSEQ, SMALLMOL, or CIF as input. Once an option is selected, the data entry boxes change to match the application (e.g., for SAXS, the protein concentration and an “advanced input” tab at the bottom allow entry of the sample container characteristics: type, thickness, density, composition). For an MX run, the crystal unit cell, the number of monomers, the number of residues per monomer, and the heavy elements per monomer are entered as well as the solvent atom composition (mM) and the solvent fraction. Input can then be manually edited using a tab at the bottom of the screen. The “Beam” and “Wedge” blocks can be similarly completed before running the program by pressing “Run”. The dose estimation results appear on the screen. Note that a full description of all the options is available in the User Guide (https://github.com/GarmanGroup/RADDOSE-3D/blob/master/doc/user-guide.pdf).

The GUI can be downloaded from https://github.com/GarmanGroup/RADDOSE-3D. To run it, Java must already be working, and if R (https://www.r-project.org/) is installed, a 3D representation of the dose distribution for the sample can be produced from the RADDOSE‐3D output.

## 5 CONCLUSIONS

In this article, we have presented three related new developments that augment the toolbox available to structural biologists faced with the challenge of RD to their samples. First, we have now incorporated an option to include an intensity decay model into the calculation of the previous “fluence weighted dose” to give a “diffraction‐decay weighted dose” and discussed the interpretation and context of this intensity decay model. Second, we have extended the capabilities of the open source RADDOSE‐3D code which originally gave dose estimates solely for incident X‐rays but which now gives the option, in its subprogram RADDOSE‐ED, of calculating the dose absorbed for incident electrons as used in MicroED experiments. Since the use of gray should allow a more realistic comparison of RD between different instruments and samples, the dose given by RADDOSE‐ED is in gray instead of $e^{-}/{Å}^{2}$, which are historically used in electron microscopy. Thirdly, we have written a RADDOSE‐3D GUI to provide a simple platform that includes options for estimating the absorbed dose for a wide range of structural biology experiments (MX, SMX, SAXS, XFEL, ED). We hope that these developments will augment the experimentalists' capabilities and contribute to a better understanding of RD effects, as well as enable further optimisation of data collection strategies.

## AUTHOR CONTRIBUTIONS

**Joshua L. Dickerson:** Conceptualization; formal analysis; investigation; methodology; project administration; software; supervision; validation; visualization; writing – original draft; writing – review and editing. **Patrick T. N. McCubbin:** Conceptualization; formal analysis; funding acquisition; investigation; methodology; software; validation; visualization; writing – original draft; writing – review and editing. **Jonathan C. Brooks‐Bartlett:** Conceptualization; formal analysis; funding acquisition; investigation; methodology; software; validation; writing – original draft; writing – review and editing. **Elspeth F. Garman:** Conceptualization; funding acquisition; project administration; supervision; resources; writing – original draft; writing – review and editing.

## FUNDING INFORMATION

We acknowledge the UK Engineering and Physical Sciences Research Council (EPSRC) for a studentship in the Systems Biology programme of the University of Oxford Doctoral Training Centre (JCBB) and for support from the EPSRC Undergraduate Vacation Placement Programme (PTNM). We are also grateful to the Collaborative Computing Project Number 4 (CCP4), UK for short‐term postdoctoral funding (JCBB).

## CONFLICT OF INTEREST STATEMENT

The authors declare no conflicts of interest.

## Supporting information


**Data S1.** Supporting Information.

## Data Availability

RADDOSE‐3D, along with the GUI and User Guide, is available at https://github.com/GarmanGroup/RADDOSE‐3D.

## References

[pro5005-bib-0001] Atakisi H , Conger L , Moreau DW , Thorne RE . Resolution and dose dependence of radiation damage in biomolecular systems. IUCrJ. 2019;6(6):1040–1053. 10.1107/S2052252519008777 PMC6830208 31709060

[pro5005-bib-0002] Baker LA , Rubinstein JL . Chapter 15 – Radiation damage in electron cryomicroscopy. Cryo‐EM part A sample preparation and data collection. In: Jensen GJ , editor. Methods in enzymology. Volume 481. USA: Academic Press; 2010. p. 371–388. 20887865 10.1016/S0076-6879(10)81015-8

[pro5005-bib-0003] Beilsten‐Edmands J , Winter G , Gildea R , Parkhurst J , Waterman D , Evans G . Scaling diffraction data in the DIALS software package: algorithms and new approaches for multi‐crystal scaling. Acta Crystallogr D Struct Biol. 2020;76(4):385–399. 32254063 10.1107/S2059798320003198 PMC7137103

[pro5005-bib-0004] Berger C , Dumoux M , Glen T , Yee NB , Mitchels JM , Patáková Z , Darrow MC, Naismith JH, Grange M. Plasma FIB milling for the determination of structures in situ. Nature. Communications. 2023;14(1):629. 10.1038/s41467-023-36372-9 PMC9902539 36746945

[pro5005-bib-0005] Bethe H . Zur Theorie des Durchgangs schneller Korpuskularstrahlen durch Materie. Ann Phys. 1930;397(3):325–400.

[pro5005-bib-0006] Bethe H . Bremsformel für Elektronen relativistischer Geschwindigkeit. Zeitschrift für Physik. 1932;76(5):293–299.

[pro5005-bib-0007] Bhattacharyya R , Dhar J , Ghosh Dastidar S , Chakrabarti P , Weiss MS . The susceptibility of disulfide bonds towards radiation damage may be explained by S…O interactions. IUCrJ. 2020;7(5):825–834. 10.1107/S2052252520008520 PMC7467163 32939274

[pro5005-bib-0008] Bieger B , Essen LO , Oesterhelt D . Crystal structure of halophilic Dodecin. Structure. 2003;11(4):375–385. 12679016 10.1016/s0969-2126(03)00048-0

[pro5005-bib-0009] Blake C , Phillips DC . Effects of X‐irradiation on single crystals of myoglobin. Proceedings of the Symposium on the Biological Effects of Ionizing Radiation at the Molecular Level; Vienna: International Atomic Energy Agency,1962. p. 183–191.

[pro5005-bib-0010] Borek D , Bromberg R , Hattne J , Otwinowski Z . Real‐space analysis of radiation‐induced specific changes with independent component analysis. J Synchrotron Radiat. 2018;25(2):451–467. 29488925 10.1107/S1600577517018148 PMC5829680

[pro5005-bib-0011] Borek D , Cymborowski M , Machius M , Minor W , Otwinowski Z . Diffraction data analysis in the presence of radiation damage. Acta Crystallogr D Biol Crystallogr. 2010;66(4):426–436. 20382996 10.1107/S0907444909040177 PMC2852307

[pro5005-bib-0012] Borek D , Dauter Z , Otwinowski Z . Identification of patterns in diffraction intensities affected by radiation exposure. J Synchrotron Radiat. 2013;20(1):37–48. 23254654 10.1107/S0909049512048807 PMC3526920

[pro5005-bib-0013] Borek D , Ginell SL , Cymborowski M , Minor W , Otwinowski Z . The many faces of radiation‐induced changes. J Synchrotron Radiat. 2007;14(1):24–33. 17211069 10.1107/S0909049506046589

[pro5005-bib-0014] Bote D , Salvat F , Jablonski A , Powell CJ . Cross sections for ionization of K, L and M shells of atoms by impact of electrons and positrons with energies up to 1GeV: analytical formulas. Atom Data Nucl Data Tabl. 2009;95(6):871–909.

[pro5005-bib-0015] Bourenkov GP , Popov AN . Optimization of data collection taking radiation damage into account. Acta Crystallogr D Biol Crystallogr. 2010;66(4):409–419. 20382994 10.1107/S0907444909054961 PMC2852305

[pro5005-bib-0016] Brice DK . Stopping powers for electrons and positrons (ICRU report 37; international commission on radiation units and measurements, Bethesda, Maryland, USA, 1984). Nucl Instrm Methods Phys Res B. 1985;12:187–188.

[pro5005-bib-0017] Brooks‐Bartlett JC . Quantifying radiation damage in X‐ray diffraction experiments in structural biology (D.Phil thesis). UK: University of Oxford, UK; 2016.

[pro5005-bib-0018] Brooks‐Bartlett JC , Batters RA , Bury CS , Lowe ED , Ginn HM , Round A , Garman EF. Development of tools to automate quantitative analysis of radiation damage in SAXS experiments. J Synchrotron Radiat. 2017;24(1):63–72. 28009547 10.1107/S1600577516015083 PMC5182020

[pro5005-bib-0019] Bury CS , Brooks‐Bartlett JC , Walsh SP , Garman EF . Estimate your dose: RADDOSE‐3D. Protein Sci. 2018;27(1):217–228. 28921782 10.1002/pro.3302 PMC5734275

[pro5005-bib-0020] Chapman HN , Caleman C , Timneanu N . Diffraction before destruction. Philos Transac R Soc B Biol Sci. 2014;369(1647):20130313. 10.1098/rstb.2013.0313 PMC4052855 24914146

[pro5005-bib-0021] Christensen J , Horton PN , Bury CS , Dickerson JL , Taberman H , Garman EF , Coles SJ. Radiation damage in small‐molecule crystallography: fact not fiction. IUCrJ. 2019;6(4):703–713. 10.1107/S2052252519006948 PMC6608633 31316814

[pro5005-bib-0022] Clabbers MTB , Abrahams JP . Electron diffraction and three‐dimensional crystallography for structural biology. Crystallogr Rev. 2018;24(3):176–204.

[pro5005-bib-0023] Clabbers MTB , Gruene T , van Genderen E , Abrahams JP . Reducing dynamical electron scattering reveals hydrogen atoms. Acta Crystallogr A. 2019;75(1):82–93. 10.1107/S2053273318013918 PMC6302931 30575586

[pro5005-bib-0024] Clabbers MTB , Shiriaeva A , Gonen T . MicroED: conception, practice and future opportunities. IUCrJ. 2022;9(2):169–179. 10.1107/S2052252521013063 PMC8895021 35371502

[pro5005-bib-0025] Colletier JP , Sawaya MR , Gingery M , Rodriguez JA , Cascio D , Brewster AS , Michels‐Clark T, Hice RH, Coquelle N, Boutet S, Williams GJ, Messerschmidt M, DePonte DP, Sierra RG, Laksmono H, Koglin JE, Hunter MS, Park H, Uervirojnangkoorn M, Bideshi DK, Brunger AT, Federici BA, Sauter NK, Eisenberg DS. De novo phasing with X‐ray laser reveals mosquito larvicide BinAB structure. Nature. 2016;539(7627):43–47. 27680699 10.1038/nature19825 PMC5161637

[pro5005-bib-0026] de la Mora E , Carmichael I , Garman EF . Effective scavenging at cryotemperatures: further increasing the dose tolerance of protein crystals. J Synchrotron Radiat. 2011;18(3):346–357. 21525642 10.1107/S0909049511007163

[pro5005-bib-0027] de la Mora E , Coquelle N , Bury CS , Rosenthal M , Holton JM , Carmichael I , Garman EF, Burghammer M, Colletier JP, Weik M. Radiation damage and dose limits in serial synchrotron crystallography at cryo‐ and room temperatures. Proc Natl Acad Sci. 2020;117(8):4142–4151. 32047034 10.1073/pnas.1821522117 PMC7049125

[pro5005-bib-0028] Dickerson JL , Garman EF . The potential benefits of using higher X‐ray energies for macromolecular crystallography. J Synchrotron Radiat. 2019;26(4):922–930. 31274414 10.1107/S160057751900612X

[pro5005-bib-0029] Dickerson JL , Garman EF . Doses for experiments with microbeams and microcrystals: Monte Carlo simulations in RADDOSE‐3D. Protein Sci. 2021;30(1):8–19. 32734633 10.1002/pro.3922 PMC7737758

[pro5005-bib-0030] Dickerson JL , Lu PH , Hristov D , Dunin‐Borkowski RE , Russo CJ . Imaging biological macromolecules in thick specimens: the role of inelastic scattering in cryoEM. Ultramicroscopy. 2022;237:113510. 35367900 10.1016/j.ultramic.2022.113510 PMC9355893

[pro5005-bib-0031] Dickerson JL , McCubbin PTN , Garman EF . RADDOSE‐XFEL: femtosecond time‐resolved dose estimates for macromolecular X‐ray free‐electron laser experiments. J Appl Cryst. 2020;53(2):549–560.

[pro5005-bib-0032] Diederichs K , McSweeney S , Ravelli RBG . Zero‐dose extrapolation as part of macromolecular synchrotron data reduction. Acta Crystallogr D Biol Crystallogr. 2003;59(5):903–909. 12777808 10.1107/s0907444903006516

[pro5005-bib-0033] Duyvesteyn HME , Kotecha A , Ginn HM , Hecksel CW , Beale EV , de Haas F , Evans G, Zhang P, Chiu W, Stuart DI. Machining protein microcrystals for structure determination by electron diffraction. Proc Natl Acad Sci. 2018;115(38):9569–9573. 30171169 10.1073/pnas.1809978115 PMC6156647

[pro5005-bib-0034] Egerton RF . Dose measurement in the TEM and STEM. Ultramicroscopy. 2021;229:113363. 34343770 10.1016/j.ultramic.2021.113363

[pro5005-bib-0035] Evans P . Scaling and assessment of data quality. Acta Crystallogr D Biol Crystallogr. 2006;62(1):72–82. 16369096 10.1107/S0907444905036693

[pro5005-bib-0036] Evans PR , Murshudov GN . How good are my data and what is the resolution? Acta Crystallogr D Biol Crystallogr. 2013;69(7):1204–1214. 23793146 10.1107/S0907444913000061 PMC3689523

[pro5005-bib-0037] Fernando NK , Cairns AB , Murray CA , Thompson AL , Dickerson JL , Garman EF , Ahmed N, Ratcliff LE, Regoutz A. Structural and electronic effects of X‐ray irradiation on prototypical [M(COD)Cl]2 catalysts. Chem A Eur J. 2021;125(34):7473–7488. 10.1021/acs.jpca.1c05759 34420303

[pro5005-bib-0038] Garman EF , Weik M . Chapter 20. In: Wlodawer A , Dauter Z , Jaskolski M , editors. Radiation damage in macromolecular crystallography. New York, NY: Springer New York; 2017. p. 467–489. 10.1007/978-1-4939-7000-1_20 28573586

[pro5005-bib-0039] Garman EF , Weik M . Radiation damage to biological macromolecules. Curr Opin Struct Biol. 2023;82:102662. 37573816 10.1016/j.sbi.2023.102662

[pro5005-bib-0040] Gati C , Bourenkov G , Klinge M , Rehders D , Stellato F , Oberthür D , Yefanov O, Sommer BP, Mogk S, Duszenko M, Betzel C, Schneider TR, Chapman HN, Redecke L. Serial crystallography on in vivo grown microcrystals using synchrotron radiation. IUCrJ. 2014;1(2):87–94. 10.1107/S2052252513033939 PMC4062088 25075324

[pro5005-bib-0041] Gerstel M , Deane CM , Garman EF . Identifying and quantifying radiation damage at the atomic level. J Synchrotron Radiat. 2015;22(2):201–212. 25723922 10.1107/S1600577515002131 PMC4344357

[pro5005-bib-0042] Glaeser RM , Downing KH . High‐resolution electron crystallography of protein molecules. Ultramicroscopy. 1993;52(3):478–486. 8116103 10.1016/0304-3991(93)90064-5

[pro5005-bib-0043] Hattne J , Shi D , Glynn C , Zee CT , Gallagher‐Jones M , Martynowycz MW , Rodriguez JA, Gonen T. Analysis of global and site‐specific radiation damage in Cryo‐EM. Structure. 2018;26(5):759–766.e4. 29706530 10.1016/j.str.2018.03.021 PMC6333475

[pro5005-bib-0044] Henderson R . The potential and limitations of neutrons, electrons and X‐rays for atomic resolution microscopy of unstained biological molecules. Q Rev Biophys. 1995;28:171–193. 7568675 10.1017/s003358350000305x

[pro5005-bib-0045] Henderson R , Unwin PNT . Three‐dimensional model of purple membrane obtained by electron microscopy. Nature. 1975;257(5521):28–32. 1161000 10.1038/257028a0

[pro5005-bib-0046] Hendrickson WA . Radiation damage in protein crystallography. J Mol Biol. 1976;106(3):889–893. 978739 10.1016/0022-2836(76)90271-0

[pro5005-bib-0047] Hirata K , Yamashita K , Ueno G , Kawano Y , Hasegawa K , Kumasaka T , Yamamoto M. ZOO: an automatic data‐collection system for high‐throughput structure analysis in protein microcrystallography. Acta Crystallogr D. 2019;75(2):138–150. 10.1107/S2059798318017795 PMC6400253 30821703

[pro5005-bib-0048] Holton JM . A beginner's guide to radiation damage. J Synchrotron Radiat. 2009;16(2):133–142. 19240325 10.1107/S0909049509004361 PMC2651760

[pro5005-bib-0049] Holton JM , Frankel KA . The minimum crystal size needed for a complete diffraction data set. Acta Crystallogr D. 2010;66(4):393–408. 20382993 10.1107/S0907444910007262 PMC2852304

[pro5005-bib-0050] Howells MR , Beetz T , Chapman HN , Cui C , Holton JM , Jacobsen CJ , Kirz J, Lima E, Marchesini S, Miao H, Sayre D, Shapiro DA, Spence JCH, Starodub D. An assessment of the resolution limitation due to radiation‐damage in X‐ray diffraction microscopy. J Electr Spectrosc. 2009;170(1–3):4–12. 10.1016/j.elspec.2008.10.008 PMC2867487 20463854

[pro5005-bib-0051] Hussein IF , Abdaljalil RO , Mohammed SA , Mkhaiber AF . Calculation of the electron stopping power of some components of human body tissues. Kuwait J Sci. 2023;50(4):545–550.

[pro5005-bib-0052] ICRU . Stopping powers for electrons and positrons. 1984. https://www.osti.gov/biblio/6965717.

[pro5005-bib-0053] Incardona MF , Bourenkov GP , Levik K , Pieritz RA , Popov AN , Svensson O . EDNA: a framework for plugin‐based applications applied to X‐ray experiment online data analysis. J Synchrotron Radiat. 2009;16(6):872–879. 19844027 10.1107/S0909049509036681

[pro5005-bib-0054] Jablonski A , Salvat F , Powell CJ , Lee AY . NIST electron elastic‐scattering cross‐section database version 4.0. Gaithersburg, MD: National Institute of Standards and Technology; 2016.

[pro5005-bib-0055] Kabsch W . XDS. Acta Crystallogr D Biol Crystallogr. 2010;66(2):125–132. 20124692 10.1107/S0907444909047337 PMC2815665

[pro5005-bib-0056] Klar PB , Krysiak Y , Xu H , Steciuk G , Cho J , Zou X , Palatinus L. Accurate structure models and absolute configuration determination using dynamical effects in continuous‐rotation 3D electron diffraction data. Nat Chem. 2023;15(6):848–855. 37081207 10.1038/s41557-023-01186-1 PMC10239730

[pro5005-bib-0057] Kmetko J , Husseini NS , Naides M , Kalinin Y , Thorne RE . Quantifying X‐ray radiation damage in protein crystals at cryogenic temperatures. Acta Crystallogr D Biol Crystallogr. 2006;62(9):1030–1038. 16929104 10.1107/S0907444906023869

[pro5005-bib-0058] Krause FF , Schowalter M , Oppermann O , Marquardt D , Müller‐Caspary K , Ritz R , Simson M, Ryll H, Huth M, Soltau H, Rosenauer A. Precise measurement of the electron beam current in a TEM. Ultramicroscopy. 2021;223:113221. 33588232 10.1016/j.ultramic.2021.113221

[pro5005-bib-0059] Krojer T , von Delft F . Assessment of radiation damage behaviour in a large collection of empirically optimized datasets highlights the importance of unmeasured complicating effects. J Synchrotron Radiat. 2011;18(3):387–397. 21525647 10.1107/S0909049511008235 PMC3083914

[pro5005-bib-0060] Langmore JP , Smith MF . Quantitative energy‐filtered electron microscopy of biological molecules in ice. Ultramicroscopy. 1992;46(1):349–373. 1336234 10.1016/0304-3991(92)90024-e

[pro5005-bib-0061] Leal RMF , Bourenkov G , Russi S , Popov AN . A survey of global radiation damage to 15 different protein crystal types at room temperature: a new decay model. J Synchrotron Radiat. 2013;20(1):14–22. 23254652 10.1107/S0909049512049114 PMC3943537

[pro5005-bib-0062] Leal RMF , Bourenkov GP , Svensson O , Spruce D , Guijarro M , Popov AN . Experimental procedure for the characterization of radiation damage in macromolecular crystals. J Synchrotron Radiat. 2011;18(3):381–386. 21525646 10.1107/S0909049511002251 PMC3268693

[pro5005-bib-0063] Li X , Zheng SQ , Egami K , Agard DA , Cheng Y . Influence of electron dose rate on electron counting images recorded with the K2 camera. J Struct Biol. 2013;184(2):251–260. 23968652 10.1016/j.jsb.2013.08.005 PMC3854003

[pro5005-bib-0064] Lucas BA , Grigorieff N . Quantification of gallium cryo‐FIB milling damage in biological lamellae. Proc Natl Acad Sci. 2023;120(23):e2301852120. 37216561 10.1073/pnas.2301852120 PMC10266028

[pro5005-bib-0065] Martynowycz MW , Clabbers MTB , Unge J , Hattne J , Gonen T . Benchmarking the ideal sample thickness in cryo‐EM. Proc Natl Acad Sci. 2021;118(49):e2108884118. 34873060 10.1073/pnas.2108884118 PMC8670461

[pro5005-bib-0066] Martynowycz MW , Zhao W , Hattne J , Jensen GJ , Gonen T . Collection of continuous rotation MicroED data from ion beam‐milled crystals of any size. Structure. 2019;27(3):545–548.e2. 30661853 10.1016/j.str.2018.12.003 PMC6476546

[pro5005-bib-0067] Masmaliyeva RC , Murshudov GN . Analysis and validation of macromolecular B values. Acta Crystallogr D Struct Biol. 2019;75(5):505–518. 31063153 10.1107/S2059798319004807 PMC6503761

[pro5005-bib-0068] McPhillips TM , McPhillips SE , Chiu HJ , Cohen AE , Deacon AM , Ellis PJ , Garman EF, Gonzalez A, Sauter NK, Phizackerley RP, Soltis SM, Kuhn P. Blu‐ice and the distributed control system: software for data acquisition and instrument control at macromolecular crystallography beamlines. J Synchrotron Radiat. 2002;9(6):401–406. 12409628 10.1107/s0909049502015170

[pro5005-bib-0069] Meents A , Gutmann S , Wagner A , Schulze‐Briese C . Origin and temperature dependence of radiation damage in biological samples at cryogenic temperatures. Proc Natl Acad Sci. 2010;107(3):1094–1099. 20080548 10.1073/pnas.0905481107 PMC2798883

[pro5005-bib-0070] Minor W , Cymborowski M , Borek D , Cooper DR , Chruszcz M , Otwinowski Z . Optimal structure determination from sub‐optimal diffraction data. Protein Sci. 2022;31(1):259–268. 34783106 10.1002/pro.4235 PMC8740829

[pro5005-bib-0071] Murray JW , Garman EF , Ravelli RBG . X‐ray absorption by macromolecular crystals: the effects of wavelength and crystal composition on absorbed dose. J Appl Cryst. 2004;37(4):513–522.

[pro5005-bib-0072] Murray JW , Rudiño‐Piñera E , Owen RL , Grininger M , Ravelli RBG , Garman EF . Parameters affecting the X‐ray dose absorbed by macromolecular crystals. J Synchrotron Radiat. 2005;12(3):268–275. 15840910 10.1107/S0909049505003262

[pro5005-bib-0073] Nass K . Radiation damage in protein crystallography at X‐ray free‐electron lasers. Acta Crystallogr D. 2019;75(2):211–218. 10.1107/S2059798319000317 PMC6400258 30821709

[pro5005-bib-0074] O'Neill P , Stevens DL , Garman EF . Physical and chemical considerations of damage induced in protein crystals by synchrotron radiation: a radiation chemical perspective. J Synchrotron Radiat. 2002;9(6):329–332. 12409618 10.1107/s0909049502014553

[pro5005-bib-0075] Owen RL , Axford D , Nettleship JE , Owens RJ , Robinson JI , Morgan AW , Dore AS, Lebon G, Tate CG, Fry EE, Ren J, Stuart DI, Evans G. Outrunning free radicals in room‐temperature macromolecular crystallography. Acta Crystallogr D Biol Crystallogr. 2012;68(7):810–818. 22751666 10.1107/S0907444912012553 PMC4791751

[pro5005-bib-0076] Owen RL , Paterson N , Axford D , Aishima J , Schulze‐Briese C , Ren J , Fry EE, Stuart DI, Evans G. Exploiting fast detectors to enter a new dimension in room‐temperature crystallography. Acta Crystallogr D Biol Crystallogr. 2014;70(5):1248–1256. 24816094 10.1107/S1399004714005379 PMC4014120

[pro5005-bib-0077] Owen RL , Rudiño‐Piñera E , Garman EF . Experimental determination of the radiation dose limit for cryocooled protein crystals. Proc Natl Acad Sci. 2006;103(13):4912–4917. 16549763 10.1073/pnas.0600973103 PMC1458769

[pro5005-bib-0078] Paithankar KS , Garman EF . Know your dose: RADDOSE. Acta Crystallogr D. 2010;66(4):381–388. 20382991 10.1107/S0907444910006724 PMC2852302

[pro5005-bib-0079] Palatinus L , Petřček V , Corrêa CA . Structure refinement using precession electron diffraction tomography and dynamical diffraction: theory and implementation. Acta Crystallogr A. 2015;71(2):235–244. 10.1107/S2053273315001266 25727873

[pro5005-bib-0080] Parkhurst JM , Crawshaw AD , Siebert CA , Dumoux M , Owen CD , Nunes P , Waterman D, Glen T, Stuart DI, Naismith JH, Evans G. Investigation of the milling characteristics of different focused‐ion‐beam sources assessed by three‐dimensional electron diffraction from crystal lamellae. IUCrJ. 2023;10(3):270–287. 10.1107/S2052252523001902 PMC10161776 36952226

[pro5005-bib-0081] Peet MJ , Henderson R , Russo CJ . The energy dependence of contrast and damage in electron cryomicroscopy of biological molecules. Ultramicroscopy. 2019;203:125–131. 30773415 10.1016/j.ultramic.2019.02.007 PMC6495108

[pro5005-bib-0082] Popov AN , Bourenkov GP . Choice of data‐collection parameters based on statistic modelling. Acta Crystallogr D Biol Crystallogr. 2003;59(7):1145–1153. 12832757 10.1107/s0907444903008163

[pro5005-bib-0083] Rodriguez JA , Ivanova MI , Sawaya MR , Cascio D , Reyes FE , Shi D , Sangwan S, Guenther EL, Johnson LM, Zhang M, Jiang L, Arbing MA, Nannenga BL, Hattne J, Whitelegge J, Brewster AS, Messerschmidt M, Boutet S, Sauter NK, Gonen T. Structure of the toxic core of α‐synuclein from invisible crystals. Nature. 2015;525(7570):486–490. 26352473 10.1038/nature15368 PMC4791177

[pro5005-bib-0084] Russo CJ , Dickerson JL , Naydenova K . Cryomicroscopy in situ: what is the smallest molecule that can be directly identified without labels in a cell? Faraday Discuss. 2022;240:277–302. 35913392 10.1039/d2fd00076h PMC9642008

[pro5005-bib-0085] Sempau J , Sánchez‐Reyes A , Salvat F , Tahar HO , Jiang SB , Fernández‐Varea JM . Monte Carlo simulation of electron beams from an accelerator head using PENELOPE. Phys Med Biol. 2001;46:1163–1186. 11324958 10.1088/0031-9155/46/4/318

[pro5005-bib-0086] Shelley KL , Garman EF . Quantifying and comparing radiation damage in the protein data Bank. Nat Commun. 2022;13(1):1314. 35288575 10.1038/s41467-022-28934-0 PMC8921271

[pro5005-bib-0087] Shi D , Nannenga BL , Iadanza MG , Gonen T . Three‐dimensional electron crystallography of protein microcrystals. Elife. 2013;nov;2:e01345. 24252878 10.7554/eLife.01345 PMC3831942

[pro5005-bib-0088] Shi Y . A glimpse of structural biology through X‐ray crystallography. Cell. 2014;159(5):995–1014. 25416941 10.1016/j.cell.2014.10.051

[pro5005-bib-0089] Spence JCH , Donatelli JJ . Inversion of dynamical Bragg intensities to complex structure factors by iterated projections. Ultramicroscopy. 2021; 222:113214. 33561601 10.1016/j.ultramic.2021.113214

[pro5005-bib-0090] Stellato F , Oberthür D , Liang M , Bean R , Gati C , Yefanov O , Barty A, Burkhardt A, Fischer P, Galli L, Kirian RA, Meyer J, Panneerselvam S, Yoon CH, Chervinskii F, Speller E, White TA, Betzel C, Meents A, Chapman HN. Room‐temperature macromolecular serial crystallography using synchrotron radiation. IUCrJ. 2014;1(4):204–212. 10.1107/S2052252514010070 PMC4107920 25075341

[pro5005-bib-0091] Sternheimer RM . The density effect for the ionization loss in various materials. Phys Rev. 1952;88:851–859.

[pro5005-bib-0092] Sternheimer RM , Berger MJ , Seltzer SM . Density effect for the ionization loss of charged particles in various substances. Atom Data Nucl Data Tabl. 1984;30(2):261–271.

[pro5005-bib-0093] Subramanian G , Basu S , Liu H , Zuo JM , Spence JCH . Solving protein nanocrystals by cryo‐EM diffraction: multiple scattering artifacts. Ultramicroscopy. 2015;148:87–93. 25461585 10.1016/j.ultramic.2014.08.013

[pro5005-bib-0094] Suzuki R , Baba S , Mizuno N , Hasegawa K , Koizumi H , Kojima K , Kumasaka T, Tachibana M. Radiation‐induced defects in protein crystals observed by X‐ray topography. Acta Crystallogr D Struct Biol. 2022;78(2):196–203. 35102885 10.1107/S205979832101281X

[pro5005-bib-0095] Sygusch J , Allaire M . Sequential radiation damage in protein crystallography. Acta Crystallogr A Crystallogr. 1988;44(4):443–448. 10.1107/s0108767388001394 2978720

[pro5005-bib-0096] Teng TY , Moffat K . Primary radiation damage of protein crystals by an intense synchrotron X‐ray beam. J Synchrotron Radiat. 2000;7(5):313–317. 16609214 10.1107/S0909049500008694

[pro5005-bib-0097] Tuijtel MW , Kreysing JP , Welsch S , Hummer G , Beck M , Turoňová B . Thinner is not always better: Optimising cryo lamellae for subtomogram averaging. bioRxiv. 2023. 10.1126/sciadv.adk6285 PMC11051657 38669330

[pro5005-bib-0098] Warkentin MA , Atakisi H , Hopkins JB , Walko D , Thorne RE . Lifetimes and spatio‐temporal response of protein crystals in intense X‐ray microbeams. IUCrJ. 2017;4(6):785–794. 10.1107/S2052252517013495 PMC5668864 29123681

[pro5005-bib-0099] Weichenberger CX , Afonine PV , Kantardjieff K , Rupp B . The solvent component of macromolecular crystals. Acta Crystallogr D Biol Crystallogr. 2015;71(5):1023–1038. 25945568 10.1107/S1399004715006045 PMC4427195

[pro5005-bib-0100] Weik M , Colletier JP . Temperature‐dependent macromolecular X‐ray crystallography. Acta Crystallogr D Biol Crystallogr. 2010;66(4):437–446. 20382997 10.1107/S0907444910002702 PMC2852308

[pro5005-bib-0101] Weik M , Kryger G , Schreurs AMM , Bouma B , Silman I , Sussman JL , Gros P, Kroon J. Solvent behaviour in flash‐cooled protein crystals at cryogenic temperatures. Acta Crystallogr D Biol Crystallogr. 2001;57(4):566–573. 11264586 10.1107/s0907444901001196

[pro5005-bib-0102] White TA , Kirian RA , Martin AV , Aquila A , Nass K , Barty A , Chapman HN. CrystFEL: a software suite for snapshot serial crystallography. J Appl Cryst. 2012;45(2):335–341.

[pro5005-bib-0103] Yang Q , Wu C , Zhu D , Li J , Cheng J , Zhang X . The reduction of FIB damage on cryo‐lamella by lowering energy of ion beam revealed by a quantitative analysis. Structure. 2023;31(10):1275–1281. 37527655 10.1016/j.str.2023.07.002

[pro5005-bib-0104] Zeldin OB , Brockhauser S , Bremridge J , Holton JM , Garman EF . Predicting the X‐ray lifetime of protein crystals. Proc Natl Acad Sci. 2013;110(51):20551–20556. 24297937 10.1073/pnas.1315879110 PMC3870734

[pro5005-bib-0105] Zeldin OB , Gerstel M , Garman EF . RADDOSE‐3D: time‐ and space‐resolved modelling of dose in macromolecular crystallography. J Appl Cryst. 2013;46(4):1225–1230.

