# Supplementary material for: Doses for X‐ray and electron diffraction: New features in RADDOSE‐3D including intensity decay models
Source: Protein Sci. 2024 Jun 25;33(7):e5005. doi: 10.1002/pro.5005 (PMC11196903; doi:10.1002/pro.5005)
Supplement: Supplementary file 1 — Data S1. Supporting Information. [file PRO-33-e5005-s001.docx]

## Supporting Information

Doses for X-ray and electron diffraction: new features in RADDOSE-3D including intensity decay models

Joshua L. Dickerson | Patrick T.N. McCubbin | Jonathan C. Brooks-Bartlett | Elspeth F. Garman

### S1.1 | Abbreviations

Table S1 lists the abbreviations used in this paper.

| MX | Macromolecular Crystallography |
| --- | --- |
| SMX | Small Molecule Crystallography |
| SAXS | Small-Angle X-ray Scattering |
| RD | Radiation Damage |
| IDM | Intensity Decay Model |
| FWD | Fluence Weighted Dose |
| DWD | Diffraction Weighted Dose |
| DDWD | Diffraction-Decay Weighted Dose |
| HEWL | Chicken Egg-White Lysozyme |
| IGD | Inverse Gamma Distribution |
| SSX | Serial Synchrotron Crystallography |
| SFX | Serial Femtosecond Crystallography |
| XFEL | X-ray Free Electron Laser |
| MicroED | Microcrystal Electron Diffraction |
| MFP | Mean Free Path |
| cryoEM | Cryogenic Electron Microscopy |
| cryoET | Cryogenic Electron Tomography |
| SPA | Single-Particle Analysis |
| FIB | Focused Ion Beam |
| ACC | Absorption Coefficient Calculation |
| GUI | Graphical User Interface |

**TABLE S1** List of abbreviations

### S1.2 | Supplementary Material for IDM section

#### S1.2.1 | Input parameters for FWD and DDWD calculations

Table S2 shows the input values used to run RADDOSE-3D for the calculation of FWD and DDWD in section 2 of the main text.

| **Input** | **Value (FWD calculation)** | **Value (DDWD calculation)** |
| --- | --- | --- |
| Crystal block: | | |
| Type | Cuboid | Cuboid |
| XYZ dimensions (μm) | 25, 15, 15 | 25, 15, 15 |
| Pixels per micron | 5 | 5 |
| AbsCoefCalc | EXP | EXP |
| PDB | 2ybh | 2ybh |
| SolventHeavyConc (mM) | Cl 1750 | Cl 1750 |
| DDM | None | LEAL |
| DECAYPARAM (γ(MGy^-1^), *B*_0_(Å^2^), *β* (Å^2^ MGy^-1^)) | N/A | 1.10, 54.2, 21.5 |
| Beam block: | | |
| Type | Experimentalpgm | Experimentalpgm |
| Pixelsize (μm, μm) | 0.05, 0.05 | 0.05, 0.05 |
| File | pseudoVoigtProfile.pgm | pseudoVoigtProfile.pgm |
| Flux (× 10^12^ photons per second) | 2.67 | 2.67 |
| Energy (keV) | 13.45 | 13.45 |
| Wedge block (exposure repeated 79 times): | | |
| Wedge (angular range in degrees to account for beam divergence) | 0–0.01 | 0–0.01 |
| ExposureTime (s) | 0.002 | 0.002 |

**TABLE S2** Input values used to run RADDOSE-3D to generate the dose estimates for the analysis shown in Figure 2. The file pseudoVoigtProfile.pgm is a generated beam profile according to a pseudo-Voigt distribution as described in the supplementary material of de la Mora et al. (2020) (i.e. with parameters FWHM in *x* = 3.0 μm, FWHM in *y* = 1.5 μm, *h*_Gauss_= 0.86, dimensions = 15 μm x 15 μm, pixel dimensions = 0.05 μm x 0.05 μm).

#### S1.2.2 | DDWD prediction for cryo-temperature sample

The DDWD was predicted for the cryo-temperature sample from de la Mora et al. (2020) using the same parameters as for the room temperature DDWD prediction with two differences. Firstly, different parameter values for the Leal et al*.* (2013) model were used since these are temperature-dependent; for the cryo-temperature prediction parameter values of, γ = 0.00748 MGy^-1^, *B*_0_ = 18.1 Å^2^, and *β =* 0.298 Å^2^ MGy^-1^ were used, as estimated using the fits shown in Figure 4 in the main text). Secondly, the DDWD estimation was extrapolated to much longer exposure times (using 10 ms exposures for efficient computation). This is to illustrate that the value of DDWD only shows a large difference from the value of FWD at these large exposure times because the intensity decay for a cryo-temperature sample is much slower. Due to the uncertainties in the estimation of such a small value of γ (there is essentially negligible decrease in the scale factor due to radiation damage that is apparent in the exposure times shown in Figure 4), this extrapolated prediction should be treated with caution.


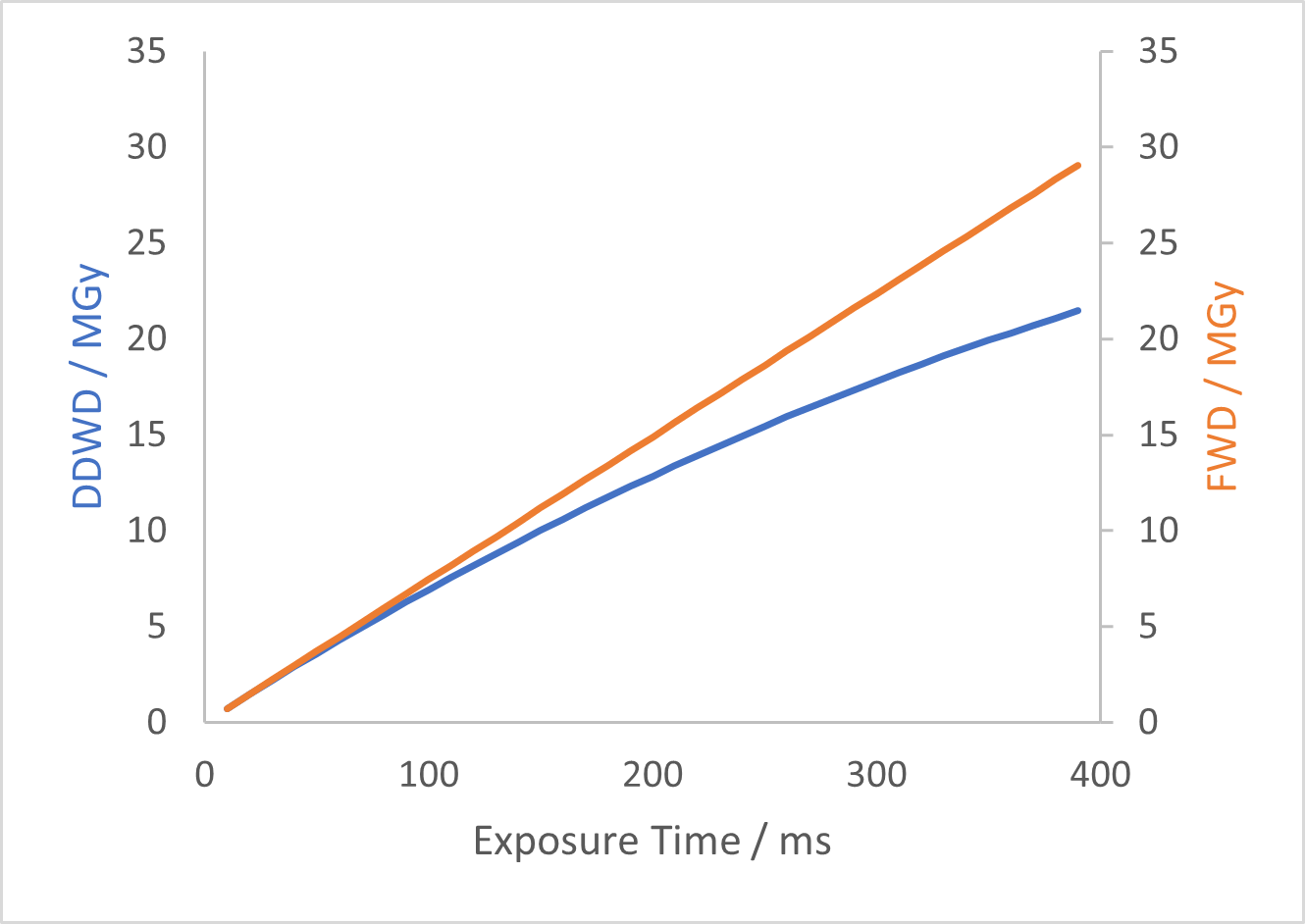


**FIGURE S1** DDWD prediction using the Leal et al. (2013) fitted to the cryo-temperature, high dose rate dataset from de la Mora et al. (2020), extrapolated to longer exposure times.

#### S1.2.3 | Details of modelling *p*_B_ in the *B_Break_* model for the scale factor term

Let us approximate the probability distribution of atomic *B*-factors as an inverse gamma distribution (IGD). Shifted IGDs are accepted as a useful approximation for atomic *B*-factor distributions (Masmaliyeva and Murshudov 2019). If this holds then the cumulative distribution function describing *P (B ≤ B_Break_)* is a regularized upper incomplete gamma function, Q:

|  | $P\left( B\leq B_{max} \right) \sim Q\left( a,\frac{b}{B_{Break}} \right)$ | (S1) |
| --- | --- | --- |

where *a* is the shape parameter, *b* is the scale parameter of the corresponding IGD, and the meaning of *B_Break_* is explained in the main text.

The scale parameter is, to a first approximation, the product of the IGD mean (which we can model as *B = B_0_ + β D )* and the shape parameter minus 1:

| $b=\left( B_{0}+\beta D \right)\left( a-1 \right)$ | (S2) |
| --- | --- |

To fit this model to the scale factor decay curve, we can fix the values of *B_0_* and *β* according to the overall *B*-factor fit (Figure 4). Under the assumptions of this model (see main text), the scale factor *K* is proportional is the ratio of the proportion *P(B ≤ B_max_)*  at a given dose to the proportion at zero dose. Hence:

| $K=const. \times\frac{Q\left( a,\frac{\left( B_{0}+\beta D \right)\left( a-1 \right)}{B_{Break}} \right)}{Q\left( a,\frac{\left( B_{0} \right)\left( a-1 \right)}{B_{Break}} \right)}$ | (S3) |
| --- | --- |

This leaves three free parameters to fit: the distribution shape (*a*), *B_Break_*, and a proportionality constant. The resulting fit to the FWD is shown in Figure 5.

The best-fit value for the shape is significantly higher than that which would be expected from theoretical arguments concerning only the translational degrees of freedom per atom (=3), and empirical fits of shifted IGDs to refined structures in the PDB (≈ 3.6) (Masmaliyeva and Murshudov 2019). To some extent this may be because the model does not incorporate any shift to the IGD: such a shift might increase with dose, but including this would introduce additional parameters making over-fitting more likely (especially because the value of  *K* at zero dose is a free parameter). Other factors affecting the *B*-factors, including vibrational modes, correlated motions, and larger scale crystal defects, may also contribute to the number of degrees of freedom.

However, inspection of Figure 5c suggests that any distribution of *p_B_* that is bell-shaped and which entirely shifts to higher *B*-factor values as dose increases would also predict a term with similar behaviour to the scale factor *K*. These conditions are similar to assumptions made when reducing the effect on diffracted intensity of *p_B_*  to that of a single average *B*-factor, and modelling this average *B*-factor to increase linearly with dose. The model can be straightforwardly adapted for a different proposed form of *p_B_* , and indeed also to a more complex boundary than a single value *B_Break_*.

#### S1.2.4 | Modelling persistent signal in diffraction images at high doses

IDMs generally predict that relative intensity should tend to zero as dose increases. However, if entire diffraction images are integrated directly, as is the case for Figure 3A of de la Mora et al. (2020), it is necessary to account for a persistent signal i.e. the relative intensity does not go to zero. To allow comparison with the models of radiation damage in de la Mora et al. (2020), we now show how the Leal *et al.* (2013) model might be extended to account for this persistent signal, again using as an example the relative intensity data from de la Mora et al. (2020). These data have been analysed through direct integration of the diffraction patterns to the edge of the detector, and are reproduced on the scatter plots in Figure S2. The persistent signal is most likely due to diffuse scattering of the beam, for example by the sample surroundings or by the solvent atoms in the crystal. In the ‘three-beam’ model used in de la Mora et al. (2020), this persistent signal was largely accounted for by the third `constant’ beam term.

It is important to stress that this persistent signal should be part of the background removed during integration of individual reflections, so only the atomic fraction causing Bragg diffraction (P_Bragg_) gives signal. Indeed, the intensity data for merged reflections of the same diffraction dataset after background subtraction, Figure 3, shows near zero persistent signal, and it was these data that are used for fitting the Leal et al*.* (2013) model in the main text. This supplementary section is solely to allow comparison with the radiation damage models in de la Mora et al. (2020).

The simplest option to model the persistent signal would be to add a horizontal asymptote to the IDM. The decay approaches this asymptote at high doses, in the style of a standard dose-response curve. Modifying the Leal et al. (2013) model in this way, we obtain:

| $M\left( D,h \right)=RI_{\infty}+\left( 1-RI_{\infty} \right)\exp\left( -\gamma^{2}D^{2} \right)\exp\left( -\frac{\left( B_{0}+\beta D \right)h^{2}}{2} \right)$ | (S4) |
| --- | --- |

where *B_0_, β* and *γ* are related to the corresponding parameters in the Leal et al*.* (2013) model but RI_∞_ is the relative intensity value of a horizontal asymptote. However, this modification is probably unsatisfactory because it does not account for the apparent slow decay of the residual signal.

Alternatively, we could extend the kinetic framework introduced in Section 2.2.2 for the Leal et al. (2013) model by positing a fraction of atoms, P_scat_, that does not contribute to Bragg diffraction, but still scatters X-rays. Radiation damage might result in the conversion of this fraction into the fraction of atoms that do not scatter or diffract (‘Scattering to None’ conversion). A possible form for the rate of this conversion is to mimic the relationship for the ‘Bragg to None’ conversion in Equation 7, giving:

| $\frac{dP_{scat}}{dD}=-2\xi^{2}DP_{scat}$ | (S5) |
| --- | --- |

where 2*ξ ^2^* is a rate constant. Thus:

| $P_{scat}=P_{0,scat}\exp\left( -\xi^{2}D^{2} \right)$ | (S6) |
| --- | --- |

where P_0,scat_  is the value of P_scat_  at zero dose. However, other forms of the rate law leading to a different power of the dose within the exponential are impossible to discount without a more systematic analysis of diffraction images at high doses. The experimental data suggest there is a weak dose-dependence to the persistent signal, but this may contain a dose-independent component e.g. due to scattering from the experimental apparatus. If we are making the assumption that the persistent signal is the result of diffuse/amorphous scattering, at such wide scattering angles as those measured during an X-ray diffraction experiment, the scattering intensity per atom in the fraction P_scat_  would therefore be weak and approximately independent of resolution. Hence:

| ${RI}_{scat}=P_{scat} A$ | (S7) |
| --- | --- |

where *A* is a constant even when *h* varies. Conversely, if the contribution of this fraction is instead due to a radiation-resistant population of Bragg scatterers, the resolution dependence would follow exp*(h^2^)* and the parameters of the RI_scat_ term in this extended model would vary with resolution. Combining Equations 6 and 9 from the main text with Equation S7 gives the following model for the relative intensity due to the two fractions P_Bragg_ and P_scat_:

| $M\left( D,h \right)=RI_{Bragg}+RI_{scat}$ | (S8) |
| --- | --- |

| $M\left( D,h \right)=RI_{0,Bragg}\exp\left( -\gamma^{2}D^{2} \right)\exp\left( -\frac{1}{2}\left( B_{0}+\beta D \right)h^{2} \right)+RI_{0,scat}\exp\left( -\xi^{2}D^{2} \right)A$ | (S9) |
| --- | --- |

where RI_0, x_ is the contribution to the relative intensity from a given fraction *x* at zero dose. If we assume that P_Bragg_ and P_scat_ are the only two fractions contributing to the intensity, it follows that RI_0,scat_ + RI_0, Bragg_ = 1, and thus:

| $M\left( D,h \right)=RI_{0,Bragg}\exp\left( -\gamma^{2}D^{2} \right)\exp\left( -\frac{1}{2}\left( B_{0}+\beta D \right)h^{2} \right)+\left( 1-RI_{0,Bragg} \right)\exp\left( -\xi^{2}D^{2} \right)A$ | (S10) |
| --- | --- |

For fitting the model, we can take *ω = ξ* ln(*A*/2) to group the redundant constants:

| $M\left( D,h \right)=RI_{0,Bragg}\exp\left( -\gamma^{2}D^{2} \right)\exp\left( -\frac{1}{2}\left( B_{0}+\beta D \right)h^{2} \right)+\left( 1-RI_{0,Bragg} \right)\exp\left( -\omega^{2}D^{2} \right)$ | (S11) |
| --- | --- |

Thus we have accounted for the persistent signal through only two parameters, RI_Bragg_ describing the proportion of the initial relative intensity due to Bragg diffraction, and *ω* describing the slow decay in the persistent signal (*ω* will vary with resolution shell if this decay is resolution-dependent). This is equivalent to the Leal *et al.* (2013) model if RI_Bragg_=1, and equivalent to the model with a horizontal asymptote (equation S4) if *ω* = 0.

Substituting into Equation 3 from the main text to evaluate the model within a given resolution range, we derive:

| $\eta= RI_{0,Bragg}\exp\left( -\gamma D^{2} \right)\frac{\int_{h_{min}}^{h_{max}} h^{2}I\exp\left( -\frac{1}{2}\left( B_{0}+\beta D \right)h^{2} \right)dh}{\int_{h_{min}}^{h_{max}} h^{2}I\exp\left( -\frac{1}{2}B_{0}h^{2} \right)dh}+\left( 1-RI_{0,Bragg} \right)\exp\left( -\omega^{2}D^{2} \right)$ | (S12) |
| --- | --- |


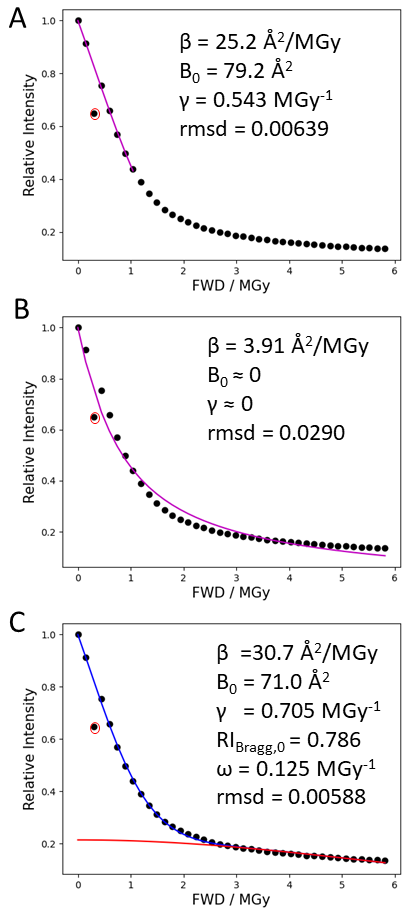


**FIGURE S2** Fits of the Leal et al. (2013) model to the room temperature high dose rate relative intensity data from de la Mora et al. (2020). (A) Fit of the Leal et al. (2013) model (magenta curve) to only the low dose region (0 to 1 MGy) of the data, which gives physically reasonable parameter values. (B) The fit of the Leal et al*.* (2013) model (magenta curve) to all the data, which gives unphysical parameter values because of the persistent intensity at high doses. (C) Fit of the model proposed in the text, giving physically reasonable parameter values when fitted against all the data. The red curve shows the contribution of the RI_scat_ term to the total relative intensity, and the blue curve shows the total relative intensity (the sum of the RI_scat_ and the RI_Bragg_ terms). The anomalously low third data point was not used during model fitting. The full range of the BEST data was used to approximate the resolution dependence of intensity decay since these relative intensities were calculated by integrating up to the edge of the detector. The values of *B_0_* and *β* are affected by this approximation, and by the fact the data are normalised against the first exposure.

Figure S2 shows the application of this model to the diffraction data from de la Mora et al. (2020). Fitting the Leal et al. (2013) model to the full data range gives non-physical parameter values. Figure S2 shows that more physically reasonable parameter values can be achieved through fitting to either the low-dose region or fitting using the new model (although these parameters are still inaccurate because the resolution-dependence of intensity was only approximated using the BEST data, as in Equation 3, integrating over the full resolution range of the BEST data, and because it is normalised against a single exposure).

Variations on this model, such as a sequential conversion from the fraction P_Bragg_ to the fraction P_scat_ and then to the fraction P_None_ could also be constructed within this framework and produce related but more complex forms of theoretical intensity decay curves. However, since equation S12, which describes parallel conversion of two distinct fractions of scatterers (in this case Bragg or diffuse) to a non-scattering fraction, has a logical theoretical basis and provided an excellent fit to the experimental decay curve, more complex models were not considered.

References

de la Mora E, Coquelle N, Bury CS, Rosenthal M, Holton JM, Carmichael I, Garman EF, Burghammer M, Colletier JP, Weik M. Radiation damage and dose limits in serial synchrotron crystallography at cryo‐ and room temperatures. Proc Natl Acad Sci. 2020;117(8):4142–4151.

Leal RMF, Bourenkov G, Russi S, Popov AN. A survey of global radiation damage to 15 different protein crystal types at room temperature: a new decay model. J Synchrotron Radiat. 2013;20(1):14–22.

Masmaliyeva RC, Murshudov GN. Analysis and validation of macromolecular B values. Acta Crystallogr D Struct Biol. 2019;75(5):505–518.
